# MAPK1 promotes the metastasis and invasion of gastric cancer as a bidirectional transcription factor

**DOI:** 10.1186/s12885-023-11480-3

**Published:** 2023-10-10

**Authors:** Yue Wang, Zheng Guo, Yueli Tian, Liang Cong, Yulu Zheng, Zhiyuan Wu, Guangle Shan, Yao Xia, Yahong Zhu, Xingang Li, Ying Song

**Affiliations:** 1 Gastroenteric Medicine and Digestive Endoscopy Center, The Second Hospital of Jilin University, Changchun, Jilin China; 2 Centre for Precision Health, Edith Cowan University, Joondalup, WA Australia; 3 Department of Bioinformatics, Thrive Bioresearch, Beijing, China; 4 School of Science, Edith Cowan University, Joondalup, WA Australia; 5 Department of Microbiology, School of Clinical Medicine, Li Ka Shing Faculty of Medicine, The University of Hong Kong, Pokfulam, Hong Kong Special Administrative Region China

**Keywords:** Gastric cancer, MAPK1, Bidirectional transcription factor, Cancer progression

## Abstract

**Background:**

The Mitogen-activated protein kinase 1 (MAPK1) has both independent functions of phosphorylating histones as a kinase and directly binding the promoter regions of genes to regulate gene expression as a transcription factor. Previous studies have identified elevated expression of MAPK1 in human gastric cancer, which is associated with its role as a kinase, facilitating the migration and invasion of gastric cancer cells. However, how MAPK1 binds to its target genes as a transcription factor and whether it modulates related gene expressions in gastric cancer remains unclear.

**Results:**

Here, we integrated biochemical assays (protein interactions and chromatin immunoprecipitation (ChIP)), cellular analysis assays (cell proliferation and migration), RNA sequencing, ChIP sequencing, and clinical analysis to investigate the potential genomic recognition patterns of MAPK1 in a human gastric adenocarcinoma cell-line (AGS) and to uncover its regulatory effect on gastric cancer progression. We confirmed that MAPK1 promotes AGS cells invasion and migration by regulating the target genes in different directions, up-regulating seven target genes (*KRT13*, *KRT6A*, *KRT81*, *MYH15*, *STARD4*, *SYTL4*, and *TMEM267*) and down-regulating one gene (*FGG*). Among them, five genes (*FGG*, *MYH15*, *STARD4*, *SYTL4*, and *TMEM267*) were first associated with cancer procession, while the other three (*KRT81*, *KRT6A*, and *KRT13*) have previously been confirmed to be related to cancer metastasis and migration.

**Conclusion:**

Our data showed that MAPK1 can bind to the promoter regions of these target genes to control their transcription as a bidirectional transcription factor, promoting AGS cell motility and invasion. Our research has expanded the understanding of the regulatory roles of MAPK1, enriched our knowledge of transcription factors, and provided novel candidates for cancer therapeutics.

**Supplementary Information:**

The online version contains supplementary material available at 10.1186/s12885-023-11480-3.

## Background

Gastric cancer ranks among the top five most common cancers and is the third leading cause of cancer-related deaths worldwide, accounting for an estimated 1 million new cases and 723,000 deaths each year annually [1]. The incidence of gastric cancer (GC) exhibits notable variations concerning sex and geographical distribution. The high incidence incidence and mortality hotspots for GC are particularly prevalent in East Asia, encompassing regions such as China and Japan, as well as in Central and South America, and Eastern Europe [2]. It is noteworthy that the susceptibility to GC is approximately twice as high in males as it is in females [3]. Numerous well-established risk factors have been associated with the development of GC, including but not limited to family history, diet habits, alcohol consumption, smoking, and infections with Helicobacter pylori and Epstein-Barr virus [2].

The classification of GC has witnessed the formulation of several systems due to diverse morphological manifestations. Notable examples include the classifications proposed by Nakamura and colleagues [4], Lauren [5], and WHO [6]. The Lauren classification holds prominence as the most commonly utilised system, while the WHO classification is hailed for its comprehensive and intricate categorisation of GC subtypes. In clinical practice, the lack of noticeable symptoms in the early stages of gastric cancer, and the fact that this disease is often mistaken for other conditions, coupled with this cancer’s characteristic to invade surrounding tissues and metastasize to distal organs, results in that gastric cancer is often diagnosed at an advanced stage [7].

Due to these well-documented epidemiological and classification factors, gastric cancer continues to exhibit a substantial mortality rate, reaching as high as 75% in various regions across the globe. This sobering statistic underscores the pressing need for further research and clinical interventions to effectively address this formidable health challenge [8]. While chemotherapy is a standard treatment for advanced gastric cancer, the median overall survival with standard chemotherapy often remains around eight months [9]. Achieving effective chemical treatment of gastric cancer relies on the inhibition of invasion and metastasis, emphasizing the importance of exploring underlying molecular processes and identifying master modulators for the development of novel targeted medications [10].

Mitogen-Activated Protein Kinase 1 (MAPK1) is an extensively studied member of the MAP kinase family, also known as extracellular signal-regulated kinases 2 (ERK2) [11, 12]. Being an essential member, MAPK1 functions in several cell signalling cascades mediating diverse cellular processes, including immune responses, proliferation, differentiation, and development [13–15]. Under normal physiological conditions, MAPK1 expression is modest and persistent in most tissues, but it undergoes significant changes in several malignancies including breast, bladder, lung, and gastric cancers [16].

Traditional in vitro tests mainly identified the role of MAPK1 in gastric cancer processes as a protein kinase. Overexpression of the *MAPK1* gene has been associated with cell migration in BGC-823 gastric cancer cells [17], while downregulation of the *MAPK1* gene significantly decreases the proliferation of MGC-803 gastric cancer cells, leading to cell cycle progression, and promote apoptosis [18]. However, MAPK1 has also been characterised as a transcription factor (TF), capable of binding to a G/CAAAG/C consensus sequence at the promoter and repressing the expression of interferon gamma-induced genes [19]. This raises the question of whether MAPK1 functions in the invasion and malignancy of gastric cancer by acting as a transcription regulator.

Transcirption factors (TFs) control chromatin and transcription by attaching to specific DNA sequences, thereby regulating gene expression and constructing to cancer development [20, 21]. Decades of research have proven that TFs with abnormal expression or functional defects can lead to uncontrolled cell proliferation and cancer development [22]. While earlier investigations into transcriptional regulation in cancer focused on TFs as transcriptional activators, recent studies have unveiled multiple alternative mechanisms of transcriptional repression in cancer. These mechanisms present potential therapeutic targets for the development of targeted anti-cancer agents [23]. Given MAPK1’s dual characteristics as both a MAP kinase and a transcription repressor, we focused on whether MAPK1 function as a transcription factor in the processes of invasion and metastasis in gastric cancer.

In our current study, we employed integrated approaches, including gene overexpression, gene knockdown, RNA sequencing (RNA-seq), and ChIP sequencing (ChIP-seq), to investigate the transcription regulatory function of MAPK1 and its effect on the procession of gastric cancer.

## Methods

### Cloning and over-expression plasmid construction

The plasmid for stable overexpression of MAPK1 was achieved by inserting the full-length coding sequence (CDS) of MAPK1 into a proper plasmid. Briefly, the MAPK1 CDS was obtained by reverse transcription-polymerase chain reaction (RT-PCR) using the complementary DNA (cDNA) of AGS cells as a template. The primers for RT-PCR were designed in terms of the gene annotation of GRCh38.p13 human genome assembly in the Ensembl database (https://asia.ensembl.org/Homo_sapiens/Info/Index). Then, the CDS was cloned into pOE3.1-CAHyg plasmid (JTS Scientific, Wuhan, China) for later experiments.

### AGS cell culture and over-expression plasmid transfection

Human gastric adenocarcinoma epithelial cell line (AGS cells, CRL-1739) from American Type Culture Collection (ATCC, Rockville, MD, USA) were cultured in Dulbecco’s Modified Eagle Medium: Nutrient Mixture F12 medium (DMEM/F12) (Gibco, GrandIsland, NY, USA) with 10% fetal bovine serum, 100 g/mL streptomycin, and 100 U/mL penicillin at 37 °C and 5% CO_2_.

Using Lipofectamine 2000 (Invitrogen, Carlsbad, CA, USA), all cell transfection was carried out following the manufacturer’s instructions. We used an empty pOE3.1-CAHyg plasmid vector as a non-targeting control to examine if the transfection reagents or the transfection process itself had any cytotoxic effects on target cells, i.e., ACS cells.

For a quantitative real-time polymerase chain reaction (qRT-PCR) investigation, the total RNA of transfected cells was extracted with TRIzol reagent (Thermo Fisher Scientific, Waltham, MA, USA) 48 h after transfection.

### Assessment of gene expression of MAPK1 by qPCR

Glyceraldehyde-3-phosphate dehydrogenase gene (*GAPDH*) was used as the control gene to assess the effects of oe-*MAPK1*. Standard protocols were used for cDNA synthesis as prescribed in previous studies [24]. Briefly, a cDNA core kit (Promega, Madison, WI, USA) was used to generate complementary DNA from 500ng of total RNA. Quantitative RT-PCR was carried out on a Bio-Rad S1000 using Hieff™ qPCR SYBR® Green Master Mix (Low Rox Plus; YEASEN, China). Table S1 displays the primer sequences. The 2^−ΔΔCT^ approach was then used to normalize the transcript concentrations to the *GAPDH* mRNA level [25].

Both over-expressed MAPK1 AGS cells and MAPK1 knockdown AGS cells were tested by this method for the assessment of MAPK1 gene expression.

### Assessment of protein expression of MAPK1 by western blot analysis

Extraction of total proteins from oe-MAPK1 AGS cells was performed using a lysis buffer (Beyotime, Shanghai, China), and the amount of protein recovered was determined with a bicinchoninic acid protein assay kit (Beyotime, Shanghai, China).

The proteins were transferred from the polyvinylidene difluoride (PVDF) membrane to a PVDF plate using sodium dodecyl sulphate polyacrylamide gel electrophoresis (SDS-PAGE) (Millipore, Bedford, MA, USA). Subsequently, the blots were cut based on the marker position prior to hybridization with antibodies during blotting, allowing the isolation of specific bands representing MAPK1 and Lamin A/C. After being rinsed in tris-buffered saline with 0.1% tween 20 detergent, the membrane was blocked for 1 h with skim milk powder (5%) before being incubated overnight with primary antibodies, i.e., anti-MAPK1 anti-GAPDH or anti-Lamin A/C (Thermo Fisher Scientific, Waltham, MA, USA) at 4 °C.

Anti-GAPDH or anti-Lamin A/C was used as the loading control antibody for this Western Blot to normalize the levels of MAPK1 protein detected by confirming that protein loading was the same across the SDS-PAGE gel.

The membrane was washed and subjected to incubation with goat anti-rabbit immunoglobulin (Ig) G secondary antibody for 1 h at room temperature, and then signals were detected using automated Tanon 4600SF chemiluminescence imaging equipment (Tanon Science & Technology, Shanghai, China).

Similar to the assessment for gene expressions, the protein expressions in both over-expressed and knockdown MAPK1 AGS cells were tested by this method.

### Transwell migration assay and invasion assay

To examine the effects of oe-MAPK1 on the migration and invasion of AGS cells establishes oe-MAPK1 cells were counted and seeded in the upper compartment of the Transwell migration and invasion chambers (Thermo Fisher Scientific, Waltham, MA, USA), respectively. The complete medium was introduced into the lower chamber in both tests. Cells from the top layer were wiped off using a cotton swab after 24 h in 5% CO_2_ at 37 °C. While the bottom chamber cells were randomly counted in three different microscope fields after being fixed with paraformaldehyde (4%), dyed with crystal violet (0.1%), and examined under a microscope.

In the invasion and migration experiments, Control, NC, and oe-MAPK1 groups were each repeated three times. The Transwell chamber with 100 µl Matrigel was used for the invasion test. All other processes were the same as for the Transwell migration assay.

### Knockdown of MAPK1 in AGS cells by siRNA

The sequences for siRNA against MAPK1 were designed using *siRNA at Whitehead* [26], a web-based tool developed by the Whitehead Institute for Biomedical Research (http://sirna.wi.mit.edu/). The sequences of siRNA and non-targeting control siRNA (siNegative RNA) were listed in Supplementary Table S1. All siRNA duplexes were purchased from Gemma (Suzhou, China).

### Library construction for RNA-seq

The RNA-seq libraries were created according to the manufacturer’s guidelines. Briefly, the whole RNA was processed with RQ1 DNase (Promega, Madison, WI, USA) to eliminate DNA contamination. Using SmartSpec Plus (Bio-Rad, Hercules, CA, USA), we measured the RNA’s absorbance at 260 and 280 nm (A260/A280) to evaluate its purity and concentration. Another method utilized the 1.5% agarose gel electrophoresis to confirm RNA purity.

NEBNext® UltraTM Directional RNA Library Prep Kit for Illumina® (New England Biolabs, Ipswich, MA, USA) was used to prepare RNA-seq libraries from 1 µg of total RNA from each sample. Double-strand cDNA was synthesized by purifying and fragmenting polyadenylated mRNAs. The DNAs were ligated to diluted NEBNext® Adaptor after end repair and “A” tailing. Single-strand cDNA was amplified, purified, quantified, and kept at -80 °C before sequencing; this was accomplished by first digesting the purified ligation products corresponding to 300–500 base-pairs (bp) with heat-labile uracil-DNA glycosylase (New England Biolabs, Ipswich, MA, USA).

After the quality control for libraries, NovaSeq 6000 system (Illumina, San Diego, CA, USA) was used to sequence in a mode of 150 nt paired-end reads.

### RNA-seq raw data clean and alignment

First, raw data longer than 2-N bases were thrown out. Raw sequencing reads were cleaned of adaptors and low-quality bases using the FASTX-Toolkit (Version 0.0.13). The study also eliminated the short reads containing less than 16 nt. Bowtie2 [27], was used to align the clean reads to the GRCh38 genome, with a maximum of four mismatches. For the purposes of counting gene reads and calculating FPKM (fragments per kilobase of transcript per million fragments mapped), only uniquely mapped reads were used [28].

### Differentially expressed gene analysis

In order to identify differentially expressed genes (DEGs), the *edgeR* package version 3.18.1 (R Bioconductor package) was employed [29]. The criterion for determining DEGs was established at a P-value for correction of < 0.05 and a fold change of > 1.5 or < 0.67, respectively.

### Chromatin immunoprecipitation sequencing for MAPK1

After 10 min of incubation at room temperature, AGS cells were cross-linked with formaldehyde (final concentration of 1%). Glycine was added to a final concentration of 0.125 M and gently mixed to halt the cross-linking reaction. On the ice, cells that had been cross-linked with formaldehyde were lysed by resuspending 10 cell pellet volumes of RIPA buffer (50 mM Tris 7.4, 150 mM NaCl, 2 mM EDTA, 0.1% SDS, 0.5% NP-40, and 0.5% deoxycholate) and pipetting them repeatedly.

Sonication of cell lysates resulted in DNA fragments of 200–500 bp being centrifuged at 12,000 g for 10 min, and the supernatant was used for immunoprecipitation without further processing.

After placing the tube containing the Pierce™ ChIP-grade Protein A/G Magnetic Bead (Thermo Fisher Scientific, Waltham, MA, USA) on the magnetic stand, the supernatant was discarded. Utilizing sodium phosphate buffer (1 mL, 0.1 M), the beads were washed thrice. 200 µL of 0.1 M sodium phosphate buffer was used to resuspend the beads. To the tube containing the beads was added 10 µg of MAPK-antibody (Cell Signaling Technologies, Danvers, MA, USA, #9102s) and normal IgG (Cell Signaling Technologies, Danvers, MA, USA, #2729s) antibody. The substance was put in a revolving wheel and kept there for 2 h at 4℃. The magnetic stand was turned on for 1 min with the tube holding the beads inside, and then the supernatant was poured out. Beads were resuspended with cell lysates. After being overnight spun at 4 °C, the sample was transferred to the magnetic stand and the supernatant was discarded. After being soaked in TE buffer (10 mM Tris [pH 8.0], 1 mM EDTA [pH 8.0]), the beads were washed five times with LiCl IP Wash Buffer (100 mM Tris [pH 7.5], 500 mM LiCl, 1% NP-40, and 0.5% deoxycholate).

The material was lyophilized, resuspended in 100 µL of Elution Buffer (100 mM NaHCO3, 1% SDS), and reverse cross-linked by incubation at 65 °C for a whole night. Purified DNA fragments were obtained using phenol extraction and ethanol precipitation after being treated with RNase A and proteinase K sequentially. 10 µL of water was used to resuspend the DNA pellet.

Following the manufacturer’s instructions, short, purified DNA fragments were end-repaired, adenylated, ligated to adaptors, and PCR amplified to create libraries using the KAPA HTP Library Preparation Kit (KAPA Biosystems, Wilmington, MA, USA, #KK8234). The libraries were created following the manufacturer’s instructions and used with the NovaSeq 6000 system (Illumina, San Diego, CA, USA) for 150 nt paired-end sequencing in high-throughput sequencing.

### ChIP-seq data analysis

Only reads that were uniquely mapped to the human GRCh38 genome after being aligned with Bowtie2 [27], were used for the following analysis. We utilized model-based analysis for ChIP-seq version 1.4 to find the binding locations of MAPK1 [30]. The background noise was assumed to be the input samples that did not undergo immunoprecipitation which was filtered by a linear signal-noise model in a previous study [31]. The peaks’ locations in relation to the transcription start site (TSS) were assigned using *DeepTools* [32], and binding profiles were visualized using this program.

To find the enriched binding motifs in peaks, we employed the Hypergeometric Optimization of Motif EnRichment (HOMER) program [33]. For visualizing the genome hits of RNA-seq and ChIP-seq, sashimi plots were created using the Integrative Genomics Viewer (IGV) (https://software.broadinstitute.org/software/igv/home) [34].

### Functional enrichment analysis

We used the KOBAS 2.0 server [35] to identify Gene Ontology (GO) terms and Kyoto Encyclopedia of Genes and Genomes (KEGG) pathways to classify DEGs and peak-associated genes according to their biological function [36]. A controlling approach based on the Benjamini-Hochberg method for the false discovery rate (FDR) statistic and the hypergeometric test were employed to determine the degree to which each term was enriched.

### Clinical data analysis

We obtained gene expression and clinical data of gastric cancer from the TCGA database (https://portal.gdc.cancer.gov) and extracted the outcome events, survival time, and target gene expression values [37]. Then we assigned gastric cancer samples to two groups (high and low expression groups), with the median of the target genes’ expression value as the cutoff value. Kaplan-Meier survival analysis and log-rank test were performed using packages (*survival 3.3.1*, *survminer*, *ggplot2 3.3.6*) in R software, version 4.1.0 (R Foundation, Austria).

### Statistical analysis

Each experiment was conducted in triplicate to estimate the mean ± standard deviation (SD). GraphPad Prism software version 8 (GraphPad PRISM, La Jolla, CA, USA) was used for the statistical analysis. To quantify the differences between the two groups, we employed the paired Student’s t-test when the homogeneity of variance assumption was satisfied between groups; otherwise, the Wilcoxon-signed rank test was used. p < 0.05 was set to indicate minimum statistical significance.

## Results

### Effect of overexpressed MAPK1 in AGS cells

To investigate the role of MAPK1 in the development of gastric cancer, we transfected AGS cells, a common human gastric cancer cell line, using the overexpression plasmid of MAPK1 (oe-MAPK1). We conducted qRT-PCR and Western blotting analysis to examine the mRNA and protein levels, respectively.

**Fig. 1:**
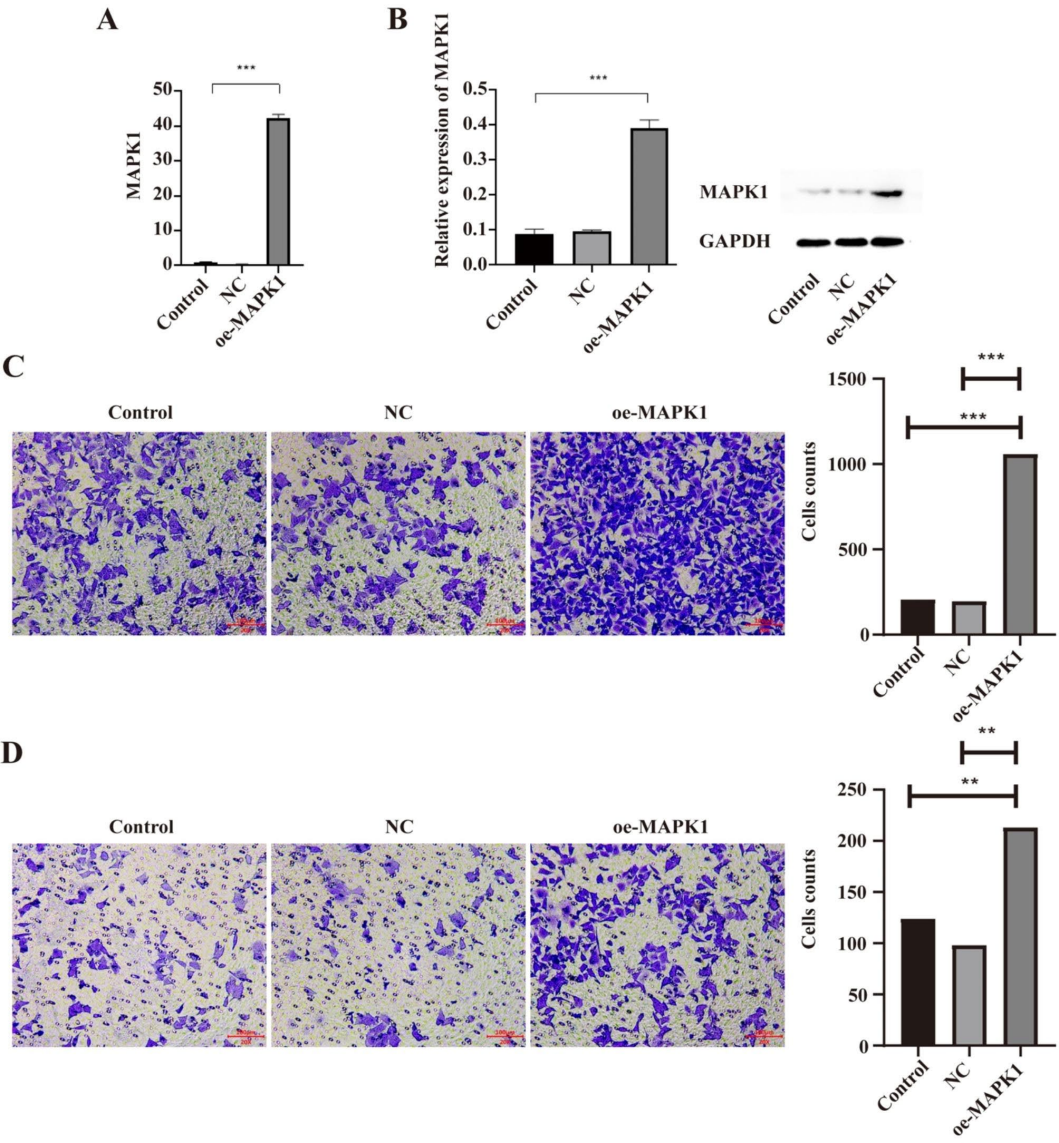
Overexpression of MAPK1 promotes migration and invasion of AGS cells. (**A**) qRT-PCR detected that significantly increased MAPK1 mRNA expression in oe-MAPK1 AGS cells compared to control cells. (**B**) Western blot analysis showed increased expression of MAPK1 in oe-MAPK1 cells compared with control cells. (**C**, **D**) In Transwell assays (20 ×), migration and invasion were increased in oe-MAPK1 AGS cells. Cells were counted in five randomly selected fields and data are presented as the means ± SEM and were evaluated using Student’s t-test (n = 3). Error bars show the mean ± SEM. **P < 0.01 and ***P < 0.001. NC: non-targeting control; oe-MAPK1: overexpression of MAPK1; SEM: standard error of the mean

In AGS cells transfected by oe-MAPK1 plasmids, results from both qRT-PCR and Western blotting analysis demonstrated a dramatically increased MAPK1 expression in case (oe-MAPK1) cells compared to control cells (P < 0.001) (Fig. 1A, B). There were no significant differences between control and non-targeting control cells, which ensured the transfection reagents, and the transfection process were non-cytotoxic to AGS cells.

Then, the function of oe-MAPK1 in cell invasion and migration was determined using transwell experiments. Oe-MAPK1 cells showed significantly induced cell proliferation and colony formation compared to control cells or non-targeting control cells. MAPK1 overexpression was shown to greatly increase the AGS cell migration (P < 0.001) and invasion (P < 0.05) (Fig. 1C, D). The non-significant differences between oe-MAPK1 cells and non-targeting control cells ensured the reliability of the experimental results.

### Transcriptome profiling of gene expression alteration in short interfering-MAPK1 AGS cells

To inspect the regulatory effect of MAPK1 in gastric cancer, we next designed three short interfering RNAs (siRNAs) (Table S1) for *MAPK1* knockdown in AGS cells followed by RNA-seq for transcriptome profiling.

**Fig. 2:**
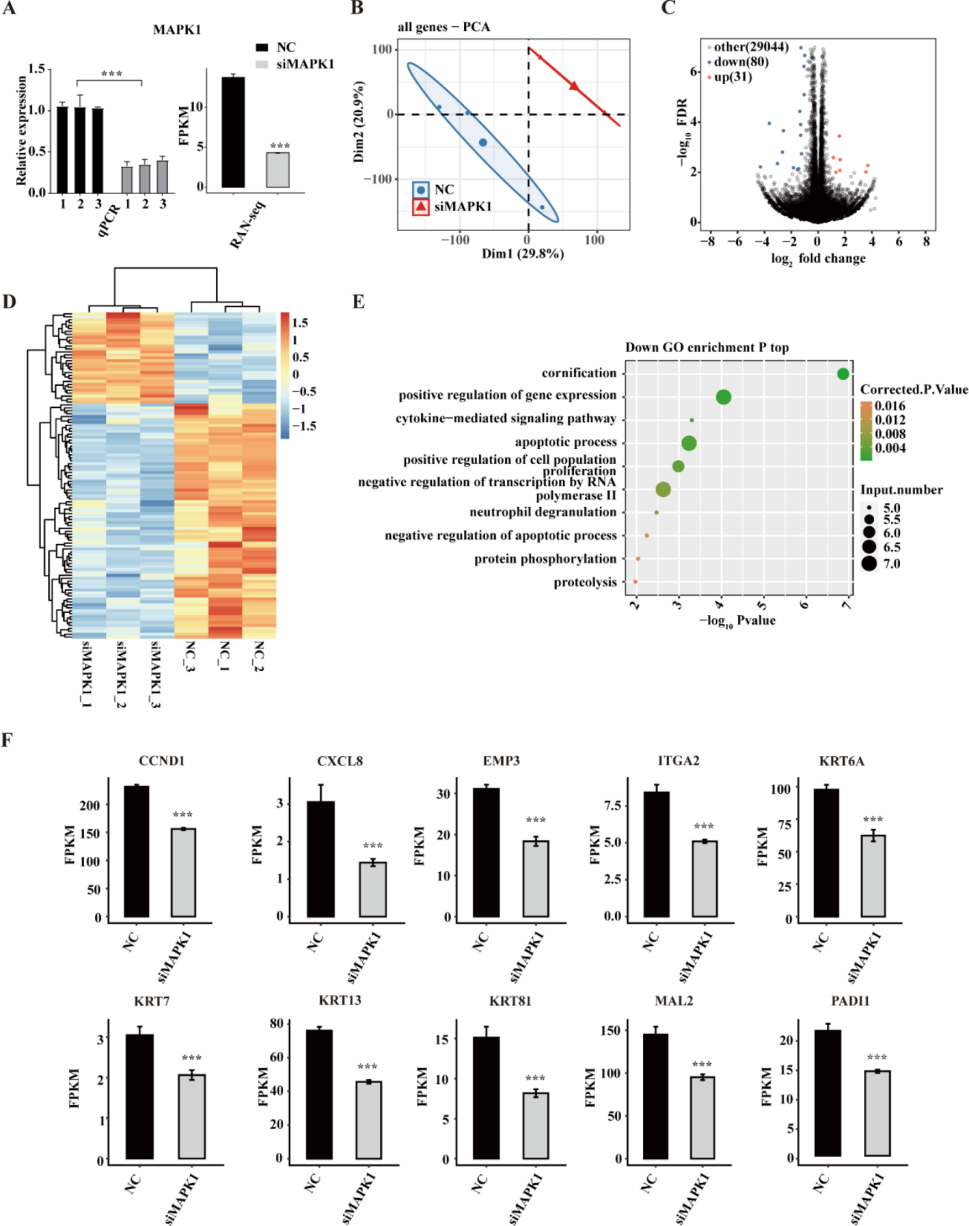
Transcriptome profiling of gene expression alteration in si-MAPK1 AGS cells. (**A**) Validation of interference effect. The effectiveness of the interference of miRNA was assessed using qRT-PCR and RNA-seq. (**B**) PCA using the FPKM value of every gene MAPK1 knockdown that was found. The confidence ellipse is the ellipse for each group. (**C**) A volcano plot displaying all DEGs that vary in expression between knockdown and NC samples. (**D**) A heat map was created using hierarchical clustering that displays the expression levels of each DEG. (**E**) Scatter plot displaying the downregulated DEGs’ enhanced GO biological process data. (**F**) A bar graph displaying the DEGs’ statistical differences and the pattern of several key genes’ expressions. Error bars show the mean ± SEM. ***P < 0.001. SEM: standard error of the mean

Three replicates of AGS cells were used for both siRNAs and non-targeting control (NC) AGS cells to generate a *MAPK1*-knockdown AGS cell model. The impact of MAPK1 regulation on gene expression was also analysed by RNA-seq. The qRT-PCR results showed that all three siRNAs significantly reduced mRNA levels of *MAPK1* (Fig. 2A) (qRT-PCT primer sequences in Table S2), indicating that the three siRNAs we designed can successfully knock down *MAPK1* in AGS cells. The siRNA1-knockdown AGS cells were then randomly chosen for subsequent experiments.

In further RNA-seq analysis, the *MAPK1* expression level in the FPKM (fragments per kilobase million) value showed a statistically significant decrease in the si-MAPK1 AGS cells compared to the NC AGS cells (Fig. 2A) (quality control results of RNA-seq in Table S3). FPKM-based principal component analysis (PCA) of all expressed genes indicated that the si-MAPK1 group and the NC group were well-separated (Fig. 2B), suggesting that the two groups could be differentiated from each other.

Through the use of the thresholds *P*-value for correction < 0.05 and fold change > 1.5 or < 0.67, *edgeR* was able to identify a total of 111 differentially expressed genes (DEGs) (Table S4, Fig. 2C), comprising 31 upregulated genes and 80 downregulated genes. The MAPK1-mediated transcription was very consistent across three independent replicates, as shown by a hierarchical clustering heat map based on all DEG expression levels (Fig. 2D). The *MAPK1* knockdown treatment drastically altered the expression of a subset of genes, as shown by these findings.

GO and KEGG enrichment analyses were conducted using these DEGs to enrich their probable biological roles. We discovered that the GO enrichment findings of downregulated genes were mostly enriched in positive regulation of gene expression, cytokine-mediated signalling pathway, apoptotic process, and positive regulation of cell population growth (Fig. 2E). Among the detected DEGs, we found 10 representative downregulated genes that were previously reported to be involved in cell proliferation and migration, i.e., *CCND1*, *CXCL8*, *EMP3*, *ITGA2*, *KRT6A*, *KRT13*, *KRT17*, *KRT81, MAL2*, and *PADI1* (Fig. 2F). These positive associations between gene expressions of MAPK1 and DEGs indicate that these genes are likely the risk factors for the migration and invasion of AGS cells. On the contrary, the significantly upregulated DEGs in knockdown MAPK1 AGS cells whose expressions are negatively associated with MAPK1, are potential protective factors.

### MAPK1 extensively interacts with genes by binding to their regulatory regions

To obtain MAPK1 binding characteristics on chromatin, we profiled the MAPK1-binding genes at the genome-wide level by ChIP-seq approach.

**Fig. 3:**
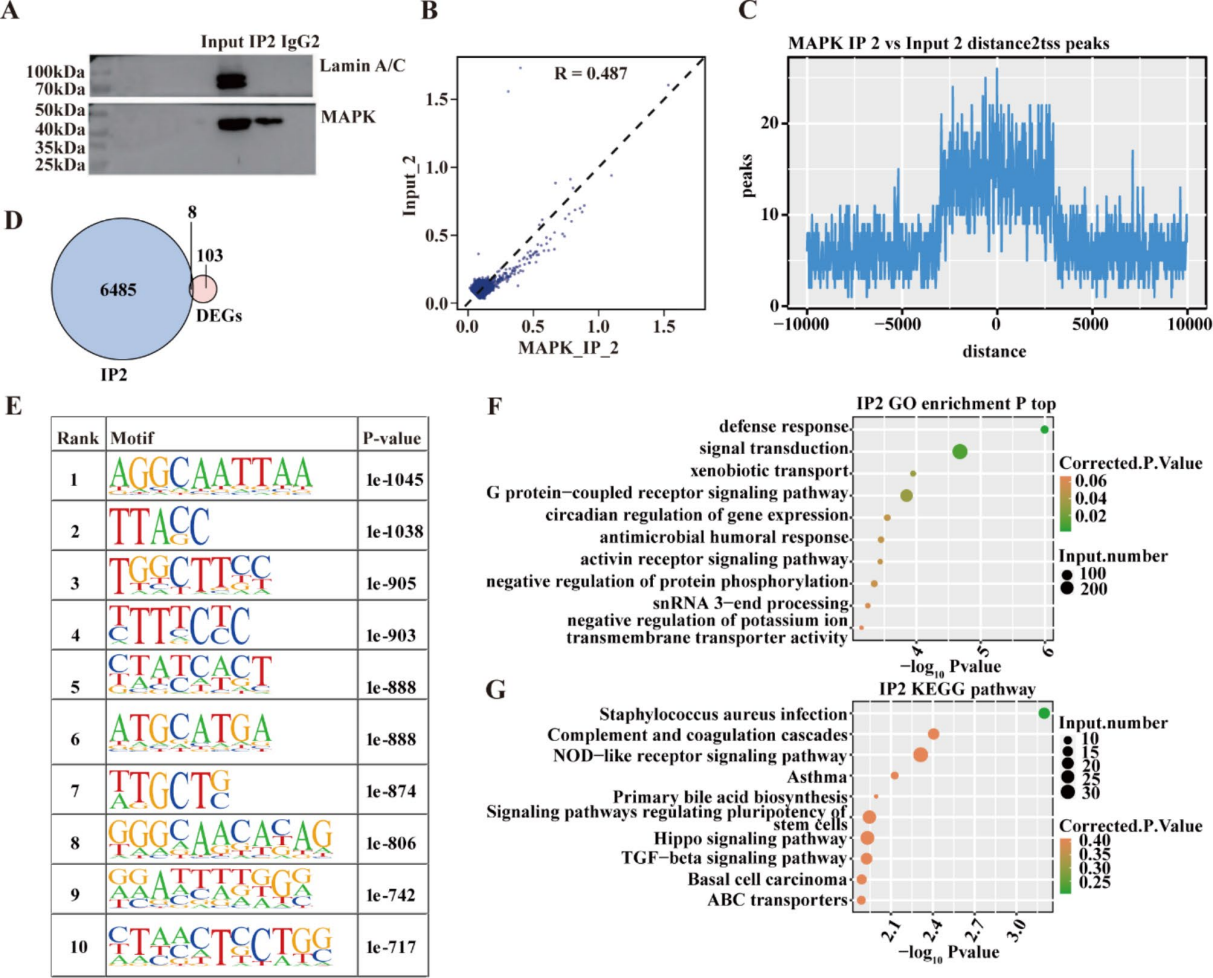
MAPK1 extensively interacts with genes by binding to their promoter region. (**A**) The Western blot result plot for MAPK1 ChIP. (**B**) Sample correlation. (**C**) ChIP-seq enrichment around TSS regions in IP2. (**D**) Venn diagram depicting overlapping genes between MAPK1 target genes and DEGs. (**E**) Motif analysis results show the enriched motifs from MAPK1 target peaks from the IP2. (**F**) A bubble plot of the top 10 enriched GO bp terms for the genes that are coupled to MAPK1. (**G**) The top 10 enriched KEGG pathways of MAPK1 target genes are shown in a bubble plot

We used MAPK1 and IgG antibodies to immunoprecipitate (IP) proteins from the lysate of AGS cells and subsequently detected them using Western Blotting analysis. Based on the data, it was determined that the MAPK1 antibody had a high degree of specificity, and MAPK1-binding DNA fragments could be effectively separated from unbound oligonucleotides (Fig. 3A). We prepared two replicates for both IP samples (IP1 and IP2) and corresponding background noises (Input1 and Input2) which were assumed not to undergo immunoprecipitation, respectively. The quality control of sequencing reads was conducted using FastQC [38], and the quality met the requirements for consequent analysis (Table S5).

Compared to the corresponding background noise (input sample), the IP sample had a considerably larger number of enriched genes, as shown by a correlation analysis between the IP2 and Input2 samples (Fig. 3B). The two replicates of experimental data showed uneven data quality. Since the peak of IP2 had more obvious enrichment near the transcription initiation site (Fig. 3C), the data of IP2 were selected for subsequent integration analysis. The ChIP-seq data of MAPK1 in AGS cells were then systematically analyzed.

A total of 92,898 peaks and 6,493 peak-associated genes were discovered using the IP2 data of ChIP-seq. After identification, we found that all these binding peaks contained one or more MAPK1 binding motifs. Through this analysis, we identified binding motifs shared by several peaks and display the 10 most drastically enhanced binding motifs (Fig. 3E) (full list in Table S6). In particular, we enriched the binding motif of G/CAAAG/C in both IP1 and IP2 samples, which was consistent with the previously reported binding motif of MAPK1 in Hela cells [19].

Through GO annotation and KEGG pathway enrichment, MAPK1 target genes were shown to be concentrated in the biological processes of defence, signal transduction, the G protein-coupled receptor signalling pathway, and the circadian control of gene expression (Fig. 3F). The NOD-like receptor signalling route, the Hippo signalling pathway, and the TGF-beta signalling pathway were among the enriched KEGG pathways (Fig. 3G).

### Identification of MAPK1 target genes in AGS cells

By integrating the RNA-seq and ChIP-seq results, overlapping analysis of MAPK1 target genes in IP2 data with DEGs after *MAPK1* knockdown obtained a total of eight target genes, whose DNA regulatory regions were bound by MAPK1 and expressions were meanwhile markedly associated with the knockdown of MAPK1 (Fig. 3D), i.e., *FGG*, *KRT6A*, *KRT13*, *KRT81*, *MYH15*, *STARD4*, *SYTL4*, and *TMEM267*. It was worth noting that MAPK1 demonstrated bidirectional regulatory functions on these genes, repressing the expression of *FGC* whilst activating the expressions of the other seven (*KRT6A*, *KRT13*, *KRT81*, *MYH15*, *STARD4*, *SYTL4*, and *TMEM267*) in AGS cells.

**Fig. 4:**
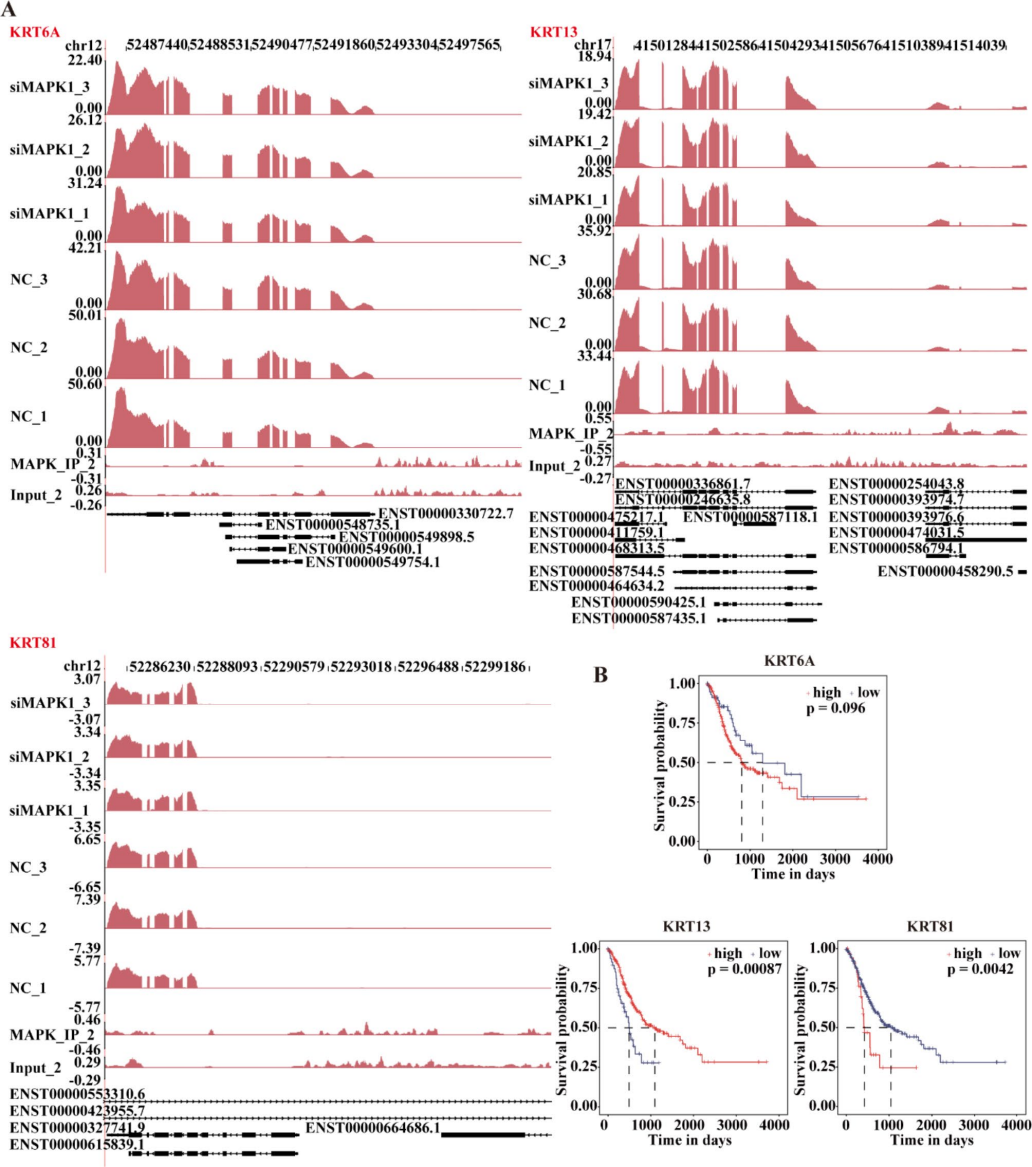
Validation of interacting genes associated with MAPK1. (**A**) The peak reads and binding locations throughout the mRNA are shown in the IGV Sashimi Plot. The red sections indicate the peak positions. The transcripts for each gene are shown below, and the read distribution of the bound gene is plotted in the top panel. (**B**) Prognostic line plots of a few significant genes from the TCGA data on gastric cancer. siMAPK1: short interfering MAPK1; NC: non-targeting control; Input: background noise without undergoing immunoprecipitation

Among them, three genes, i.e., *KRT6A*, *KRT13*, and *KRT81*, were related to cancer metastasis and migration. We next used an integrative genomics viewer (IGV) [39] to visualise the peak reads and binding locations throughout the mRNA by Sashimi Plot for these three genes (Fig. 4A). Our results indicated that MAPK1 may activate these three target genes (*KRT81*, *KRT6A*, and *KRT13)* by binding to their promotor regions.

Additionally, survival analysis was conducted in the TCGA gastric cancer database to further assess the clinical importance of these eight target genes in gastric cancer. Kaplan-Meier survival analysis of two subgroups based on the expressions of six genes (*FGG*, *KRT6A*, *KRT13*, *KRT81*, *MYH15*, and *TMEM267*) predicted distinct survial outcomes (Fig. 4B, Fig S1, S2 and S3). Patient outcomes were not significantly influenced by expressions of *STARD4* and *SYTL4* (Fig S4 and S5).

## Discussion

Several types of cancer have been shown to have aberrant MAPK1 expression, mounting evidence that MAPK1 is linked to cancer invasion and metastasis [40–42]. In prostate cancer cells, for instance, TGF-β therapy leads to nuclear accumulation of MAPK1, which in turn promotes cancer cell EMT and upregulates the production of MMP1, MMP2/9, and MMP9 [43]. To facilitate cancer cell migration and decrease adhesion, transcription factor Snail in breast cancer may stimulate nuclear translocation of phosphorylated MAPK [44]. The involvement of MAPK in mediating extracellular matrix activity may alter gastric cancer cell invasion and migration. The MAPK/ERK pathways have been shown to play a role in controlling gastric cancer cell migration and invasion induced by IL-22, PRL3, NAIF1, and CCDC134 [45]. Inhibiting gastric cancer growth by competitive binding to MEK1, as the MAPK1-109aa expressed by *circMAPK1*, is one of the mechanisms by which it works to deactivate MAPK1 and its downstream components in the MAPK pathway [46]. However, previous research has concentrated on MAPK1 as a kinase that controls cancer’s ability to invade and spread. Its function as a transcription regulator by DNA binding activity in cancer cells and the role it plays in the invasion and spread of cancer are mostly unknown.

One of the hallmarks of cancer is its tendency to metastasize [9]. Tumour cell invasiveness, epithelial-mesenchymal transition features, tumour cell-cancer microenvironment communication, and extracellular matrix remodelling all play a role in tumour metastasis [47–49]. Metastasis is therefore a leading cause of mortality for people with gastric cancer. MAPK1 is an essential oncogene that stimulates gastric cancer’s cell proliferation, migration, and invasion and an increased level of MAPK1 expression is associated with a higher risk of developing gastric cancer [50]. Our study shows that AGS cell invasion and migration are dramatically facilitated by MAPK1 overexpression. As a result, we postulated that MAPK1 promotes gastric cancer cell metastasis and invasion through its gene-binding function, ability to combine the promoter area of target genes, effects on the target genes expression, and effects on protein level.

Against the above backdrop, we used *MAPK1* knockdown in AGS cells and RNA-seq to test this theory. Our study identified the risk and protective gene sets for the migration and invasion of AGS cells. Of a total of 111 DEGs, 80 of them were significantly downregulated and 31 were upregulated. GO enrichment analysis reveals that the majority of downregulated genes are involved in activities including promoting gene expression, preventing apoptosis, and positively regulating cell proliferation. Ten typical genes involved in cellular metastasis are among the genes that were repressed here (Fig. 2F). Thus, MAPK1 shows the ability to increase AGS cell invasion and migration by activating these risk genes.

Next, we used ChIP-seq to investigate the MAPK1 binding genes at a genome-wide scale. We found that 6,493 genes had 92,898 binding peaks. Together, the findings from the ChIP-seq and RNA-seq analyses revealed eight genes whose expression is controlled by MAPK1 and whose binding to MAPK1 was previously unknown. *KRT81*, *KRT6A*, and *KRT13* are examples of these genes that have been linked to cancer metastasis and migration in humans [51–55]. In summary, ChIP-seq combined with RNA-seq revealed that MAPK1 selectively binds to the promoter regions of *KRT81*, *KRT6A*, and *KRT13* genes and regulates their transcription. This function of MAPK1 in regulating gene expression may be related to cancer invasion and metastasis. These results provide credence to the idea that MAPK1 promotes cancer cell invasion and migration via binding to the promoter regions of *KRT81*, *KRT6A*, and *KRT13*.

Over 1800 TFs are being identified in the human genome [21]. MAPK1 showed its role as a transcription repressor besides protein kinases in a previous study using experiments in Hela cells, and the DNA binding motif of MAPK1 in this cell model was identified as G/CAAAG/C [19]. The present study validates this DNA binding motif of MAPK1 in AGS cells, by which MAPK1 represses the target gene (*FGC*) (Fig. 3E). Furthermore, our study contributes a list of novel DNA binding motifs for MAPK1 in gastric cancer and demonstrates that MAPK1 can function as a bidirectional transcription factor that activates and represses the transcriptions of different target genes in gastric cancer cells. This finding supports the emerging views that many TFs have opposite regulatory effects on different genes relying on differentiating local binding motifs and recruiting multiple cofactors available in different environments [56, 57]. Identifying the tissue-specific and cell-specific functions of transcription factors and understanding their functions is important for understanding the comprehensive molecular regulations underlying cell proliferation and cancer progression [58, 59]. The new mechanism of MAPK1 as a bidirectional transcription factor provides a theoretical basis for the development of combined drugs to inhibit MAPK1 activity from multiple perspectives, offering a promising avenue for targeted GC treatment. Additionally, the identification of target genes regulated by MAPK1 in this study unveils novel candidate molecules with potential therapeutic implications for GC.

Recent developments in experimental technologies, including RNA-seq and ChIP-seq, are revolutionized appoaches for identifying DNA binding sites for a TF of interest [60, 61]. However, several limitations in the present study need to be discussed. First, ChIP-seq provides information on the genomic regions that are bound by a specific protein, but the resolution of the technique is limited by the size of the DNA fragments that are generated during the experiment. This can make it difficult to precisely locate binding sites within larger genomic regions. Secondly, the technical variability can influence the mapping results of TF binding sites, for example, differences in the quality of the antibodies used for immunoprecipitation, variations in sample preparation, and differences in sequencing depth. Being focused on a single-cell line, the current study may limit its potential impacton on the generalisability and applicability of our findinds to other GC cell lines or tissues. Conducting further research involving a variety of GC cell lines and tissues are needed in the future to enhance the broader relevance of current results. In addition, restrictions in the efficiency of the immunoprecipitation step, or the fact that some regions may be inaccessible in terms of chromatin structure can lead to incomplete coverage for binding sites investigation. Last but not the least, ChIP-seq provides information on the genomic regions that are bound by a specific protein, but it does not provide information on the protein-protein interactions that may be involved in regulating gene expression. The limited information on protein-protein interactions, especially on the factors collaborating with a master transcription regulator, appeals to more systematic studies.

## Conclusions

In conclusion, our findings show that MAPK1 knockdown significantly increases invasiveness and migration in gastric cancer cells. MAPK1 can function in gastric cancer metastasis and invasion as a bidirectional transcription factor. Furthermore, elevated levels of MAPK1 target genes in gastric cancer patients can be bound to the promoter regions to increase or decrease their expression, thereby facilitating gastric cell migration and invasion.

### Electronic supplementary material

Below is the link to the electronic supplementary material.

Supplementary Material 1

Supplementary Material 2

## Data Availability

The RNA-seq and ChIP-seq datasets generated in this study are available at the Gene Expression Ominbus (GEO, https://www.ncbi.nlm.nih.gov/geo/) repository accession number GSE234020 and GSE234022. The gene expression and clinical data of gastric cancer are publicly available in the TCGA database (https://portal.gdc.cancer.gov). Other datasets used and/or analyzed during the current study will be made available upon reasonable request from the corresponding authors.

## Abbreviations

AGS cells: A human gastric adenocarcinoma cell-line
CA: California
CCDC134: Coiled-coil domain containing 134
CCND1: Cyclin D1
CDS: coding sequence
ChIP: Chromatin immunoprecipitation
CXCL8: C-X-C motif chemokine ligand 8
DEG: Differentially expressed gene
DNA: Deoxyribonucleic acid
EDTA: Ethylenediaminetetraacetic acid
EMP3: Epithelial membrane protein 3
ERK2: Eextracellular signal-regulated kinases 2
FDR: False discovery rate
FGG: Fibrinogen gamma chain
FPKM: Fragments per kilobase million
GAPDH: Glyceraldehyde-3-phosphate dehydrogenase
GO: Gene Ontology
IL-22: Interleukin 22
ITGA2: Integrin subunit alpha 2
KEGG: Kyoto Encyclopedia of Genes and Genomes
KOBAS: KEGG Orthology-Based Annotation System
KRT: Keratin
MA: Massachusetts
MAL2: Mal, T cell differentiation protein 2
MAPK1: Mitogen-activated protein kinase 1
MD: Maryland
MEK: Meiotic chromosome-axis-associated kinase
MYH15: Myosin heavy chain 15
NAIF1: Nuclear apoptosis inducing factor 1
NOD: Nucleotide binding oligomerization domain
PAD: Peptidylarginine deiminase
PCA: Principal component analysis
PRL3: Protein-tyrosine phosphatase of regenerating liver 3
qRT-PCR: Quantitative reverse transcription polymerase chain reaction
SDS-PAGE: Sodium dodecyl sulphate polyacrylamide gel electrophoresis
SEM: Standard error of the mean
siRNAs: Short interfering ribonucleic acid
STARD4: Steroidogenic acute regulatory protein-related lipid transfer domain containing 4
SYTL4: Synaptotagmin like 4
TGF-beta: Transforming growth factor beta
TMEM267: Transmembrane protein 267
TSS: Transcription start site
USA: United States of America
WI: Wisconsin
