# Supplementary material for: MAPK1 promotes the metastasis and invasion of gastric cancer as a bidirectional transcription factor
Source: BMC Cancer. 2023 Oct 10;23:959. doi: 10.1186/s12885-023-11480-3 (PMC10563293; doi:10.1186/s12885-023-11480-3)
Supplement: Supplementary file 1 — Supplementary Material 1 [file 12885_2023_11480_MOESM1_ESM.docx]

| **Supplementary Table S1**. Sequences of siRNAs | | |
| --- | --- | --- |
| **Name** | **Sense (5'-3')** | **Antisense (5'-3')** |
| siRNA1 (MAPK1-Homo-355) | GUGCUCUGCUUAUGAUAAUTT | AUUAUCAUAAGCAGAGCACTT |
| siRNA2 (MAPK1-Homo-513) | CACCAACCAUCGAGCAAAUTT | AUUUGCUCGAUGGUUGGUGTT |
| siRNA3 (MAPK1-Homo-714) | CCACCUGUGAUCUCAAGAUTT | AUCUUGAGAUCACAGGUGGTT |
| Non-targeting control siRNA | UUCUCCGAACGUGUCACGU | ACGUGACACGUUCGGAGAA |

| **Supplementary Table S2.** Primer sequences for RT-PCR | | |
| --- | --- | --- |
| Gene | Primer | Sequence |
| MAPK1 | Forward (5'-3') | ATTACGACCCGAGTGACGAG |
|  | Reverse (3'-5') | CCTGGCTGGAATCTAGCAGT |
| GAPDH | Forward (5'-3') | GGTCGGAGTCAACGGATTTG |
|  | Reverse (3'-5') | GGAAGATGGTGATGGGATTTC |

| **Supplementary Table S3.** Quality control for RNA-Seq | | | | | | | | | | | |
| --- | --- | --- | --- | --- | --- | --- | --- | --- | --- | --- | --- |
| **SampleID** | **raw_total** | **clean_total** | **ratio_total** | **raw_base** | **clean_base** | **ratio_base** | **uniqtag** | **Q20** | **Q30** | **GC** | **DUP** |
| NC_1 | 55228412 | 52367713 | 94.82% | 8.284G | 7.149G | 86.29% | 11527377(43.21%) | 98.64 | 95.89 | 50% | 25.87% |
| NC_2 | 97905030 | 92768241 | 94.75% | 14.686G | 12.631G | 86.01% | 19236797(40.52%) | 98.65 | 95.88 | 50% | 23.31% |
| NC_3 | 86326418 | 82279012 | 95.31% | 12.949G | 11.121G | 85.88% | 16515867(39.44%) | 98.68 | 95.95 | 51% | 21.97% |
| siMAPK1_1 | 86590388 | 81335742 | 93.93% | 12.989G | 11.027G | 84.90% | 17187360(40.98%) | 98.66 | 95.88 | 50% | 23.22% |
| siMAPK1_2 | 81914208 | 77772093 | 94.94% | 12.287G | 10.915G | 88.83% | 14815882(37.37%) | 98.69 | 95.95 | 51% | 22.76% |
| siMAPK1_3 | 121937672 | 115769384 | 94.94% | 18.291G | 15.531G | 84.91% | 24030579(40.66%) | 98.68 | 95.95 | 50% | 21.33% |

| **Supplementary Table S4**. Differentially expressed genes between MAPK1-knockdown cells and non-targeting control cells | | | | | | | | | | | | | | |
| --- | --- | --- | --- | --- | --- | --- | --- | --- | --- | --- | --- | --- | --- | --- |
|  | | | | | | | | | | | | | | |
| **Gene Symbol** | **Chr** | **baseMean** | **log_2_FC** | **log_2_FCSE** | **stat** | **pvalue** | **padj** | **NC_1** | **NC_2** | **NC_3** | | **siMAPK1_1** | **siMAPK1_2** | **siMAPK1_3** |
| SRPK3 | chrX | 4.88 | 3.70 | 1.33 | 2.79 | 5.31E-03 | 1.00E+00 | 0.00 | 0.01 | 0.00 | 0.06 | | 0.07 | 0.04 |
| AMELX | chrX | 4.45 | 3.56 | 1.38 | 2.59 | 9.65E-03 | 1.00E+00 | 0.00 | 0.06 | 0.00 | 0.18 | | 0.53 | 0.20 |
| SNORD17 | chr20 | 21.44 | 1.64 | 0.55 | 2.95 | 3.13E-03 | 1.00E+00 | 1.21 | 1.48 | 1.27 | 1.56 | | 7.26 | 3.87 |
| CTB-52I2.4 | chr19 | 12.84 | 1.62 | 0.61 | 2.64 | 8.34E-03 | 1.00E+00 | 0.06 | 0.12 | 0.14 | 0.35 | | 0.39 | 0.30 |
| TMEM202-AS1 | chr15 | 26.00 | 1.58 | 0.44 | 3.57 | 3.57E-04 | 1.00E+00 | 0.11 | 0.20 | 0.12 | 0.49 | | 0.49 | 0.35 |
| MYH16 | chr7 | 19.05 | 1.33 | 0.51 | 2.58 | 9.98E-03 | 1.00E+00 | 0.06 | 0.07 | 0.02 | 0.16 | | 0.12 | 0.13 |
| ETV7 | chr6 | 32.29 | 1.13 | 0.38 | 3.01 | 2.59E-03 | 1.00E+00 | 0.28 | 0.20 | 0.29 | 0.60 | | 0.55 | 0.57 |
| ACTG2 | chr2 | 221.46 | 1.03 | 0.16 | 6.26 | 3.80E-10 | 6.36E-08 | 1.04 | 1.04 | 1.39 | 2.56 | | 2.14 | 2.50 |
| PPP1R36 | chr14 | 44.70 | 0.89 | 0.33 | 2.71 | 6.66E-03 | 1.00E+00 | 0.44 | 0.25 | 0.26 | 0.57 | | 0.55 | 0.60 |
| GANAB | chr11 | 12370.19 | 0.83 | 0.05 | 16.24 | 2.84E-59 | 7.86E-56 | 51.27 | 54.91 | 54.88 | 96.29 | | 96.63 | 97.56 |
| HABP4 | chr9 | 1668.07 | 0.80 | 0.07 | 11.02 | 3.06E-28 | 4.24E-25 | 9.40 | 8.89 | 8.89 | 15.72 | | 15.08 | 16.95 |
| TPM4 | chr19 | 27481.92 | 0.79 | 0.05 | 16.36 | 3.62E-60 | 1.33E-56 | 49.46 | 49.43 | 49.11 | 89.22 | | 83.67 | 86.81 |
| CTD-3148I10.9 | chr19 | 49.40 | 0.78 | 0.30 | 2.60 | 9.23E-03 | 1.00E+00 | 1.63 | 1.80 | 1.48 | 2.64 | | 2.65 | 3.25 |
| ZNF345 | chr19 | 60.31 | 0.77 | 0.28 | 2.77 | 5.53E-03 | 1.00E+00 | 0.34 | 0.28 | 0.24 | 0.52 | | 0.42 | 0.53 |
| ODAM | chr4 | 452.54 | 0.75 | 0.12 | 6.13 | 8.96E-10 | 1.32E-07 | 6.97 | 5.39 | 6.56 | 10.67 | | 9.83 | 11.51 |
| OGFR | chr20 | 4589.47 | 0.72 | 0.07 | 9.72 | 2.38E-22 | 2.40E-19 | 20.62 | 22.71 | 23.69 | 33.47 | | 39.94 | 38.76 |
| MEX3D | chr19 | 843.20 | 0.71 | 0.09 | 7.97 | 1.60E-15 | 7.07E-13 | 5.79 | 6.84 | 6.02 | 10.63 | | 10.24 | 10.19 |
| CHST14 | chr15 | 739.86 | 0.68 | 0.09 | 7.44 | 1.01E-13 | 3.40E-11 | 8.12 | 7.79 | 7.92 | 12.58 | | 13.73 | 12.30 |
| FGD3 | chr9 | 368.00 | 0.66 | 0.12 | 5.33 | 9.76E-08 | 8.64E-06 | 1.37 | 1.45 | 1.62 | 2.25 | | 2.61 | 2.31 |
| UPK3B | chr7 | 254.07 | 0.65 | 0.17 | 3.92 | 8.70E-05 | 2.53E-03 | 2.00 | 2.14 | 2.82 | 3.52 | | 4.44 | 3.26 |
| PAGE4 | chrX | 151.86 | 0.64 | 0.21 | 3.10 | 1.94E-03 | 2.90E-02 | 2.08 | 1.31 | 1.48 | 2.11 | | 2.35 | 3.11 |
| KLF2 | chr19 | 537.95 | 0.64 | 0.11 | 5.66 | 1.52E-08 | 1.60E-06 | 5.31 | 4.03 | 4.31 | 6.78 | | 7.48 | 7.11 |
| LINC01996 | chr17 | 195.84 | 0.64 | 0.16 | 3.86 | 1.13E-04 | 3.09E-03 | 3.66 | 3.14 | 4.18 | 5.58 | | 5.46 | 6.21 |
| XXbac-BPG181B23.9 | chr6 | 132.16 | 0.63 | 0.19 | 3.26 | 1.11E-03 | 1.87E-02 | 4.33 | 6.59 | 5.94 | 8.88 | | 8.75 | 9.30 |
| THAP7-AS1 | chr22 | 100.68 | 0.63 | 0.23 | 2.76 | 5.79E-03 | 1.00E+00 | 0.67 | 0.79 | 0.87 | 1.41 | | 0.94 | 1.34 |
| LMTK3 | chr19 | 797.65 | 0.63 | 0.10 | 6.16 | 7.23E-10 | 1.08E-07 | 2.91 | 3.27 | 3.09 | 4.36 | | 5.62 | 4.67 |
| FGG | chr4 | 151.25 | 0.63 | 0.17 | 3.60 | 3.24E-04 | 7.06E-03 | 0.96 | 1.12 | 0.92 | 1.54 | | 1.56 | 1.62 |
| TUBA5P | chr1 | 119.69 | 0.62 | 0.21 | 2.99 | 2.79E-03 | 1.00E+00 | 0.75 | 1.13 | 1.00 | 1.34 | | 1.67 | 1.54 |
| ZNF488 | chr10 | 167.89 | 0.61 | 0.17 | 3.65 | 2.58E-04 | 5.88E-03 | 1.13 | 1.23 | 1.07 | 1.91 | | 1.79 | 1.64 |
| PALM3 | chr19 | 197.90 | 0.59 | 0.16 | 3.62 | 3.00E-04 | 6.63E-03 | 1.70 | 1.50 | 1.95 | 2.39 | | 2.79 | 2.67 |
| CEL | chr9 | 268.89 | 0.59 | 0.14 | 4.09 | 4.32E-05 | 1.40E-03 | 2.50 | 2.91 | 2.78 | 3.60 | | 4.71 | 4.23 |
| LINC02747 | chr11 | 429.41 | -0.59 | 0.12 | -5.06 | 4.19E-07 | 3.09E-05 | 7.06 | 5.83 | 6.56 | 4.52 | | 4.39 | 4.20 |
| PADI1 | chr1 | 2864.19 | -0.59 | 0.07 | -7.94 | 2.05E-15 | 8.75E-13 | 18.89 | 22.66 | 22.21 | 14.18 | | 14.94 | 13.99 |
| KRT7 | chr12 | 335.32 | -0.59 | 0.14 | -4.35 | 1.39E-05 | 5.73E-04 | 2.87 | 2.81 | 3.47 | 2.02 | | 2.29 | 1.88 |
| CCND1 | chr11 | 30300.37 | -0.59 | 0.04 | -13.19 | 9.97E-40 | 2.21E-36 | 231.38 | 238.24 | 226.35 | 153.32 | | 159.66 | 155.77 |
| SNRK | chr3 | 226.69 | -0.59 | 0.17 | -3.45 | 5.52E-04 | 1.09E-02 | 1.21 | 1.45 | 0.90 | 0.87 | | 0.74 | 0.78 |
| STARD4 | chr5 | 126.18 | -0.60 | 0.22 | -2.73 | 6.33E-03 | 6.88E-02 | 1.06 | 0.77 | 0.62 | 0.64 | | 0.49 | 0.48 |
| ASAP2 | chr2 | 1071.68 | -0.60 | 0.10 | -6.08 | 1.19E-09 | 1.69E-07 | 5.67 | 6.05 | 4.49 | 3.79 | | 3.57 | 3.48 |
| SWSAP1 | chr19 | 114.29 | -0.60 | 0.22 | -2.72 | 6.57E-03 | 1.00E+00 | 2.03 | 2.30 | 2.98 | 1.53 | | 2.00 | 1.42 |
| FAXDC2 | chr5 | 97.43 | -0.60 | 0.23 | -2.60 | 9.43E-03 | 1.00E+00 | 0.52 | 0.53 | 0.49 | 0.44 | | 0.34 | 0.26 |
| VDR | chr12 | 94.53 | -0.61 | 0.21 | -2.84 | 4.57E-03 | 1.00E+00 | 0.57 | 0.63 | 0.62 | 0.43 | | 0.39 | 0.40 |
| NFATC2 | chr20 | 263.53 | -0.61 | 0.14 | -4.47 | 7.98E-06 | 3.59E-04 | 1.07 | 1.30 | 1.12 | 0.78 | | 0.76 | 0.79 |
| UGCG | chr9 | 609.77 | -0.61 | 0.12 | -4.92 | 8.68E-07 | 5.58E-05 | 5.38 | 5.51 | 3.83 | 3.56 | | 3.07 | 3.10 |
| MIGA1 | chr1 | 206.51 | -0.61 | 0.16 | -3.84 | 1.22E-04 | 3.25E-03 | 0.83 | 0.74 | 0.68 | 0.41 | | 0.54 | 0.53 |
| AC006262.6 | chr19 | 248.93 | -0.63 | 0.14 | -4.38 | 1.18E-05 | 5.02E-04 | 1.24 | 1.21 | 1.39 | 0.83 | | 0.93 | 0.78 |
| MAL2 | chr8 | 13516.56 | -0.63 | 0.07 | -8.79 | 1.49E-18 | 1.10E-15 | 150.05 | 157.75 | 126.94 | 101.58 | | 93.08 | 90.30 |
| LGALS7B | chr19 | 110.15 | -0.63 | 0.24 | -2.65 | 8.00E-03 | 1.00E+00 | 4.51 | 5.45 | 8.42 | 3.63 | | 4.06 | 4.43 |
| TNFAIP3 | chr6 | 313.68 | -0.63 | 0.12 | -5.07 | 3.93E-07 | 2.92E-05 | 2.43 | 2.23 | 2.24 | 1.58 | | 1.48 | 1.45 |
| UHMK1 | chr1 | 1203.86 | -0.64 | 0.19 | -3.36 | 7.85E-04 | 1.42E-02 | 5.47 | 5.41 | 3.56 | 3.18 | | 3.05 | 3.17 |
| TRNP1 | chr1 | 1141.94 | -0.64 | 0.08 | -8.50 | 1.97E-17 | 1.15E-14 | 21.67 | 21.68 | 20.60 | 13.96 | | 13.36 | 14.32 |
| SLC41A1 | chr1 | 1857.33 | -0.64 | 0.06 | -9.96 | 2.22E-23 | 2.46E-20 | 10.61 | 10.80 | 10.85 | 7.05 | | 6.92 | 7.04 |
| ZNF10 | chr12 | 110.26 | -0.65 | 0.22 | -2.93 | 3.39E-03 | 1.00E+00 | 0.82 | 0.72 | 0.68 | 0.52 | | 0.34 | 0.56 |
| GASAL1 | chr8 | 79.58 | -0.65 | 0.25 | -2.63 | 8.53E-03 | 1.00E+00 | 0.78 | 1.11 | 0.91 | 0.72 | | 0.54 | 0.57 |
| CDA | chr1 | 92.40 | -0.65 | 0.23 | -2.87 | 4.08E-03 | 1.00E+00 | 3.86 | 4.22 | 4.46 | 2.10 | | 2.88 | 3.10 |
| MSMO1 | chr4 | 2618.05 | -0.65 | 0.18 | -3.72 | 2.00E-04 | 4.77E-03 | 39.01 | 36.06 | 27.22 | 24.59 | | 21.88 | 19.31 |
| TMEM267 | chr5 | 128.74 | -0.66 | 0.24 | -2.72 | 6.48E-03 | 7.01E-02 | 1.37 | 2.06 | 1.10 | 1.17 | | 1.05 | 0.72 |
| EXPH5 | chr11 | 224.65 | -0.67 | 0.17 | -3.89 | 9.93E-05 | 2.82E-03 | 0.96 | 0.65 | 0.68 | 0.49 | | 0.54 | 0.42 |
| KRT6A | chr12 | 7677.48 | -0.67 | 0.08 | -8.26 | 1.50E-16 | 8.02E-14 | 90.69 | 103.86 | 98.32 | 68.98 | | 64.21 | 53.91 |
| KDM1B | chr6 | 177.63 | -0.67 | 0.17 | -3.91 | 9.34E-05 | 2.69E-03 | 1.43 | 1.12 | 1.01 | 0.73 | | 0.74 | 0.77 |
| SYTL4 | chrX | 508.28 | -0.67 | 0.10 | -6.49 | 8.71E-11 | 1.72E-08 | 2.72 | 2.63 | 2.31 | 1.59 | | 1.65 | 1.62 |
| GPRC5B | chr16 | 378.35 | -0.68 | 0.11 | -6.03 | 1.68E-09 | 2.27E-07 | 2.09 | 2.13 | 2.15 | 1.37 | | 1.31 | 1.35 |
| MITF | chr3 | 96.93 | -0.69 | 0.22 | -3.10 | 1.93E-03 | 1.00E+00 | 0.58 | 0.55 | 0.40 | 0.30 | | 0.35 | 0.31 |
| ABAT | chr16 | 466.92 | -0.71 | 0.11 | -6.25 | 4.20E-10 | 6.84E-08 | 2.04 | 1.64 | 1.76 | 1.16 | | 1.08 | 1.11 |
| CFL2 | chr14 | 679.70 | -0.72 | 0.10 | -6.88 | 6.12E-12 | 1.50E-09 | 5.72 | 5.47 | 4.39 | 3.30 | | 3.13 | 3.16 |
| BIRC3 | chr11 | 646.68 | -0.72 | 0.10 | -7.09 | 1.30E-12 | 3.60E-10 | 3.65 | 3.19 | 2.92 | 1.98 | | 2.09 | 1.92 |
| NDUFC2 | chr11 | 306.80 | -0.73 | 0.15 | -4.94 | 7.67E-07 | 5.03E-05 | 5.79 | 5.50 | 3.90 | 3.26 | | 2.92 | 3.04 |
| RP11-680H20.2 | chr11 | 57.04 | -0.73 | 0.28 | -2.60 | 9.31E-03 | 1.00E+00 | 0.46 | 0.39 | 0.36 | 0.30 | | 0.22 | 0.22 |
| ITGA2 | chr5 | 1749.48 | -0.74 | 0.08 | -9.34 | 9.54E-21 | 8.80E-18 | 8.53 | 9.27 | 7.45 | 5.01 | | 5.34 | 4.96 |
| IL6ST | chr5 | 384.60 | -0.75 | 0.15 | -5.09 | 3.67E-07 | 2.74E-05 | 1.54 | 1.72 | 1.08 | 0.94 | | 0.83 | 0.85 |
| PKIB | chr6 | 84.94 | -0.75 | 0.27 | -2.74 | 6.18E-03 | 1.00E+00 | 0.98 | 1.08 | 0.76 | 0.76 | | 0.59 | 0.36 |
| KRT13 | chr17 | 5823.38 | -0.76 | 0.06 | -12.31 | 7.67E-35 | 1.41E-31 | 75.56 | 72.65 | 80.35 | 44.58 | | 47.73 | 44.68 |
| NCEH1 | chr3 | 683.96 | -0.76 | 0.09 | -8.10 | 5.51E-16 | 2.77E-13 | 5.49 | 5.80 | 5.00 | 3.34 | | 3.30 | 3.10 |
| EMP3 | chr19 | 1335.01 | -0.78 | 0.09 | -9.01 | 2.09E-19 | 1.66E-16 | 32.26 | 29.06 | 32.04 | 16.61 | | 20.47 | 17.99 |
| C15orf62 | chr15 | 54.38 | -0.80 | 0.30 | -2.66 | 7.78E-03 | 1.00E+00 | 0.97 | 0.68 | 0.89 | 0.41 | | 0.63 | 0.44 |
| CYB5R4 | chr6 | 242.62 | -0.81 | 0.16 | -4.94 | 7.90E-07 | 5.11E-05 | 1.03 | 1.05 | 0.84 | 0.70 | | 0.49 | 0.50 |
| SSC4D | chr7 | 159.21 | -0.81 | 0.17 | -4.82 | 1.46E-06 | 8.31E-05 | 2.35 | 2.16 | 2.06 | 1.19 | | 1.25 | 1.32 |
| RP11-401P9.7 | chr16 | 70.84 | -0.83 | 0.26 | -3.12 | 1.80E-03 | 1.00E+00 | 0.97 | 0.83 | 0.66 | 0.42 | | 0.58 | 0.40 |
| CCK | chr3 | 64.92 | -0.84 | 0.29 | -2.93 | 3.38E-03 | 1.00E+00 | 1.26 | 1.26 | 1.16 | 0.46 | | 0.99 | 0.64 |
| PDE5A | chr4 | 86.66 | -0.86 | 0.23 | -3.70 | 2.14E-04 | 1.00E+00 | 0.40 | 0.37 | 0.30 | 0.22 | | 0.17 | 0.20 |
| NPIPB3 | chr16 | 262.63 | -0.87 | 0.16 | -5.33 | 9.94E-08 | 8.73E-06 | 2.17 | 2.21 | 2.17 | 0.91 | | 1.55 | 1.18 |
| STEAP1 | chr7 | 42.58 | -0.88 | 0.32 | -2.73 | 6.31E-03 | 1.00E+00 | 0.99 | 1.09 | 1.15 | 0.56 | | 0.72 | 0.53 |
| XKR9 | chr8 | 95.98 | -0.89 | 0.24 | -3.71 | 2.06E-04 | 1.00E+00 | 1.10 | 1.04 | 0.75 | 0.61 | | 0.57 | 0.41 |
| GNE | chr9 | 63.56 | -0.91 | 0.28 | -3.28 | 1.03E-03 | 1.00E+00 | 0.52 | 0.36 | 0.42 | 0.18 | | 0.26 | 0.26 |
| SELENOT | chr3 | 2097.68 | -0.92 | 0.08 | -12.09 | 1.26E-33 | 2.00E-30 | 20.97 | 21.07 | 18.36 | 11.65 | | 10.06 | 10.69 |
| KRT81 | chr12 | 737.38 | -0.92 | 0.12 | -7.79 | 6.60E-15 | 2.44E-12 | 12.41 | 16.24 | 16.72 | 8.76 | | 8.53 | 7.19 |
| HIPK3 | chr11 | 265.18 | -0.93 | 0.17 | -5.57 | 2.59E-08 | 2.63E-06 | 1.66 | 1.23 | 1.00 | 0.71 | | 0.64 | 0.71 |
| DMRT1 | chr9 | 38.45 | -0.93 | 0.33 | -2.78 | 5.38E-03 | 1.00E+00 | 0.50 | 0.45 | 0.46 | 0.26 | | 0.22 | 0.28 |
| TMEM156 | chr4 | 159.21 | -0.96 | 0.17 | -5.55 | 2.90E-08 | 2.84E-06 | 2.34 | 2.51 | 2.59 | 1.42 | | 1.33 | 1.16 |
| S100A3 | chr1 | 41.70 | -0.97 | 0.32 | -3.01 | 2.64E-03 | 1.00E+00 | 2.00 | 2.06 | 1.94 | 0.95 | | 0.91 | 1.22 |
| BCAS1 | chr20 | 34.23 | -0.98 | 0.37 | -2.63 | 8.55E-03 | 1.00E+00 | 0.12 | 0.17 | 0.21 | 0.07 | | 0.11 | 0.09 |
| KLK14 | chr19 | 370.10 | -0.98 | 0.12 | -8.03 | 9.84E-16 | 4.74E-13 | 12.14 | 12.24 | 13.10 | 5.99 | | 7.21 | 6.17 |
| SERPINB2 | chr18 | 565.67 | -1.00 | 0.10 | -10.20 | 1.91E-24 | 2.34E-21 | 8.10 | 8.49 | 8.40 | 4.37 | | 4.26 | 4.10 |
| KLK5 | chr19 | 148.67 | -1.01 | 0.19 | -5.18 | 2.17E-07 | 1.78E-05 | 2.84 | 4.01 | 4.02 | 1.80 | | 2.12 | 1.64 |
| IGFL1 | chr19 | 83.92 | -1.05 | 0.23 | -4.47 | 7.90E-06 | 1.00E+00 | 4.19 | 4.10 | 4.89 | 2.25 | | 2.33 | 1.93 |
| UBD | chr6 | 125.91 | -1.06 | 0.21 | -4.99 | 5.93E-07 | 4.12E-05 | 5.90 | 4.69 | 6.76 | 2.99 | | 2.25 | 3.13 |
| CXCL8 | chr4 | 164.97 | -1.10 | 0.20 | -5.46 | 4.85E-08 | 4.55E-06 | 3.41 | 3.61 | 2.14 | 1.63 | | 1.40 | 1.30 |
| CTD-2020K17.3 | chr17 | 32.28 | -1.24 | 0.40 | -3.12 | 1.82E-03 | 1.00E+00 | 0.82 | 0.58 | 0.42 | 0.17 | | 0.28 | 0.31 |
| CCL22 | chr16 | 82.58 | -1.29 | 0.24 | -5.32 | 1.06E-07 | 1.00E+00 | 1.40 | 1.19 | 1.09 | 0.41 | | 0.51 | 0.58 |
| MYH15 | chr3 | 34.78 | -1.30 | 0.36 | -3.59 | 3.33E-04 | 1.00E+00 | 0.18 | 0.19 | 0.22 | 0.07 | | 0.07 | 0.10 |
| ITGAX | chr16 | 43.13 | -1.31 | 0.34 | -3.84 | 1.24E-04 | 1.00E+00 | 0.27 | 0.34 | 0.18 | 0.10 | | 0.12 | 0.10 |
| TNFRSF9 | chr1 | 46.45 | -1.37 | 0.33 | -4.13 | 3.69E-05 | 1.00E+00 | 0.31 | 0.29 | 0.36 | 0.16 | | 0.08 | 0.13 |
| ADAMTS14 | chr10 | 1681.99 | -1.38 | 0.07 | -18.94 | 4.88E-80 | 2.70E-76 | 12.87 | 13.49 | 13.32 | 5.02 | | 5.57 | 4.92 |
| CAVIN4 | chr9 | 17.64 | -1.47 | 0.55 | -2.69 | 7.15E-03 | 1.00E+00 | 0.31 | 0.31 | 0.15 | 0.07 | | 0.06 | 0.14 |
| RN7SL368P | chr19 | 15.00 | -1.54 | 0.58 | -2.66 | 7.91E-03 | 1.00E+00 | 3.64 | 1.95 | 1.74 | 1.00 | | 1.04 | 0.53 |
| MAPK1 | chr22 | 1836.48 | -1.69 | 0.07 | -24.67 | 2.39E-134 | 2.64E-130 | 14.18 | 14.16 | 12.96 | 4.36 | | 4.39 | 4.23 |
| RP11-75C10.6 | chr17 | 11.02 | -1.84 | 0.68 | -2.72 | 6.60E-03 | 1.00E+00 | 0.18 | 0.29 | 0.17 | 0.10 | | 0.05 | 0.03 |
| DENND2C | chr1 | 12.59 | -2.59 | 0.70 | -3.69 | 2.20E-04 | 1.00E+00 | 0.11 | 0.05 | 0.11 | 0.01 | | 0.00 | 0.03 |
| RP11-226M10.3 | chr17 | 8.53 | -2.66 | 0.84 | -3.16 | 1.57E-03 | 1.00E+00 | 0.19 | 0.29 | 0.17 | 0.07 | | 0.00 | 0.03 |
| LINC02404 | chr12 | 5.71 | -3.04 | 1.07 | -2.84 | 4.49E-03 | 1.00E+00 | 0.16 | 0.26 | 0.18 | 0.06 | | 0.02 | 0.00 |
| ORM2 | chr9 | 9.11 | -3.64 | 0.94 | -3.86 | 1.12E-04 | 1.00E+00 | 0.32 | 0.68 | 1.03 | 0.00 | | 0.04 | 0.11 |
| CDKN2B-AS1 | chr9 | 3.39 | -4.30 | 1.57 | -2.74 | 6.09E-03 | 1.00E+00 | 0.02 | 0.01 | 0.00 | 0.00 | | 0.00 | 0.00 |

| **Supplementary Table S5**. Quality control for ChIP-Seq | | | | | | | | | | | |
| --- | --- | --- | --- | --- | --- | --- | --- | --- | --- | --- | --- |
| **SampleID** | **raw_total** | **clean_total** | **ratio_total** | **raw_base** | **clean_base** | **ratio_base** | **uniqtag** | **Q20** | **Q30** | **GC** | **DUP** |
| Input_1 | 45029406 | 44074281 | 97.88% | 6.754G | 6.418G | 95.02% | 17677661(79.91%) | 98.5 | 95.52 | 47% | 79.51% |
| Input_2 | 78943264 | 77463967 | 98.13% | 11.841G | 11.255G | 95.05% | 29999627(77.20%) | 98.55 | 95.53 | 41% | 77.86% |
| MAPK_IP_1 | 56886356 | 55083651 | 96.83% | 8.533G | 8.047G | 94.30% | 21844997(78.87%) | 98.56 | 95.51 | 43% | 79.76% |
| MAPK_IP_2 | 41595396 | 40785543 | 98.05% | 6.239G | 5.954G | 95.43% | 16181229(78.96%) | 98.57 | 95.55 | 43% | 80.03% |

| **Supplementary Table S6**. Homer de novo Motif Results | | | | | | | |
| --- | --- | --- | --- | --- | --- | --- | --- |
| **Rank** | **Motif** | **P-value** | **log P-value** | **% of Targets** | **% of Background** | **STD**  **(BgSTD)** | **Best Match/Details** |
| 1 | 5'-AGGCAATTAA-3' | 1e-1045 | -2.41E+03 | 55.65% | 43.00% | 102.9bp (160.4bp) | Hmx1/MA0896.1/Jaspar(0.821) |
| 2 | 5'-TTA[C/G]C-3' | 1e-1038 | -2.39E+03 | 71.72% | 59.65% | 102.2bp (169.4bp) | REB1/MA0363.1/Jaspar(0.848) |
| 3 | 5'-TGG[C/T]TTC[C/T]-3' | 1e-905 | -2.09E+03 | 49.60% | 37.94% | 104.3bp (168.7bp) | GCR1/MA0304.1/Jaspar(0.770) |
| 4 | 5'-[T/C]TT[T/C]C[T/C]C-3' | 1e-903 | -2.08E+03 | 58.10% | 46.33% | 104.1bp (170.4bp) | PRDM1/MA0508.3/Jaspar(0.853) |
| 5 | 5'-CTA[T/C]CACT-3' | 1e-888 | -2.05E+03 | 67.57% | 56.18% | 102.6bp (163.7bp) | At5g04390(C2H2)/col200-At5g04390-DAP-Seq(GSE60143)/Homer(0.777) |
| 6 | 5'-ATGCATGA-3' | 1e-888 | -2.05E+03 | 58.63% | 46.97% | 103.2bp (167.3bp) | ASD-1(RRM)/Caenorhabditis_elegans-RNCMPT00180-PBM/HughesRNA(0.890) |
| 7 | 5'-[T/A]TGCT[G/C]-3' | 1e-874 | -2.01E+03 | 79.15% | 68.88% | 100.5bp (184.1bp) | POL010.1_DCE_S_III/Jaspar(0.843) |
| 8 | 5'-[G/T]GGCAACA[C/T/G]AG-3' | 1e-806 | -1.86E+03 | 60.02% | 48.93% | 101.1bp (176.6bp) | kni/dmmpmm(Papatsenko)/fly(0.692) |
| 9 | 5'-[G/A/T][G/A/C]A[T/A/C]TTT[G/T/A][G/A/C]-3' | 1e-742 | -1.71E+03 | 68.62% | 58.29% | 102.1bp (164.7bp) | ATHB21(HB)/colamp-ATHB21-DAP-Seq(GSE60143)/Homer(0.639) |
| 10 | 5'-[C/T/A]T[A/T/C][A/C]CT[C/G/T]CTG[G/C]-3' | 1e-717 | -1.65E+03 | 63.21% | 52.83% | 101.3bp (168.2bp) | SRSF2(RRM)/Homo_sapiens-RNCMPT00072-PBM/HughesRNA(0.672) |
| 11 | 5'-CTC[T/A][A/T][G/C/A][C/A]-3' | 1e-651 | -1.50E+03 | 68.72% | 59.07% | 102.6bp (174.8bp) | z/dmmpmm(SeSiMCMC)/fly(0.743) |
| 12 | 5'-T[G/C][C/G]CA[A/T/G]-3' | 1e-639 | -1.47E+03 | 58.49% | 48.60% | 102.1bp (176.9bp) | NFIA/MA0670.1/Jaspar(0.968) |
| 13 | 5'-[T/G]T[T/G][C/T]G[T/G/C][T/C]T[T/C/G][T/C][T/C]-3' | 1e-613 | -1.41E+03 | 40.02% | 30.80% | 102.3bp (158.2bp) | VRN1(ABI3VP1)/col-VRN1-DAP-Seq(GSE60143)/Homer(0.788) |
| 14 | 5'-[T/C]C[A/T/C]CA[C/G/T/A]TTC[T/A/C][A/T/G/C]-3' | 1e-595 | -1.37E+03 | 44.35% | 35.04% | 104.6bp (167.2bp) | PU.1-IRF(ETS:IRF)/Bcell-PU.1-ChIP-Seq(GSE21512)/Homer(0.716) |
| 15 | 5'-[A/T]AATG[A/C]-3' | 1e-586 | -1.351E+03 | 54.53% | 44.87% | 105.1bp  (153.8bp) | AT2G31460(REMB3)/col-AT2G31460-  DAP-Seq(GSE60143)/Homer(0.869) |
| 16 | 5'-[T/G]GG[A/T/C/G][A/G]GA[A/T]G[G/T/A]-3' | 1e-563 | -1.30E+03 | 27.35% | 19.58% | 102.8bp (181.0bp) | REF2(RRM)/Drosophila_melanogaster-RNCMPT00059-PBM/HughesRNA(0.701) |
| 17 | 5'-[T/C]AGT[A/T/G/C]CT-3' | 1e-509 | -1.17E+03 | 54.04% | 45.20% | 103.7bp (159.7bp) | SPL11(SBP)/col100-SPL11-DAP-Seq(GSE60143)/Homer(0.788) |
| 18 | 5'-GATCTTG-3' | 1e-508 | -1.17E+03 | 39.17% | 30.80% | 102.0bp (174.1bp) | MATR3(RRM)/Homo_sapiens-RNCMPT00037-PBM/HughesRNA(0.849) |
| 19 | 5'-[C/T/G][C/A]AAACCATAT[T/C/G/A]-3' | 1e-415 | -9.57E+02 | 29.12% | 22.22% | 103.5bp (155.3bp) | HuR(RRM)/Homo_sapiens-RNCMPT00136-PBM/HughesRNA(0.791) |
| 20 | 5'-CATGATCCAATC-3' | 1e-381 | -8.79E+02 | 51.30% | 43.67% | 102.5bp (166.5bp) | AARE(HLH)/mES-cMyc-ChIP-Seq/Homer(0.757) |
| 21 | 5'-GC[G/C][A/C][G/C]A-3' | 1e-368 | -8.49E+02 | 65.31% | 57.98% | 103.0bp (172.9bp) | ZBTB33/MA0527.1/Jaspar(0.781) |
| 22 | 5'-[G/A/T]CA[T/G][C/T]C[T/A]-3' | 1e-338 | -7.80E+02 | 23.59% | 17.82% | 102.7bp (181.9bp) | G3BP2(RRM)/Homo_sapiens-RNCMPT00021-PBM/HughesRNA(0.798) |
| 23 | 5'-[G/C/A]A[G/A][A/T]T[C/T/G]TG[G/A][G/A/T]TG-3' | 1.00E-275 | -6.34E+02 | 4.75% | 2.44% | 99.8bp (177.9bp) | GATA11/MA1014.1/Jaspar(0.670) |
| 24 | 5'-[T/G/A][A/G/T][G/A]TGA[G/A]TGA[G/A]T-3' | 1.00E-260 | -6.01E+02 | 12.13% | 8.37% | 103.1bp (157.5bp) | WUS1(Homeobox)/colamp-WUS1-DAP-Seq(GSE60143)/Homer(0.815) |
| 25 | 5'-CACCATGTGAA-3' | 1.00E-231 | -5.33E+02 | 4.80% | 2.63% | 102.9bp (186.8bp) | INO4/INO4_YPD/4-INO4,37-INO2(Harbison)/Yeast(0.829) |
| 26 | 5'-[C/T]ACA[C/T][A/T/G][C/T][A/T/G]C3' | 1.00E-228 | -5.25E+02 | 11.29% | 7.88% | 102.1bp (166.0bp) | Rbm24(RRM)/Tetraodon_nigroviridis-RNCMPT00285-PBM/HughesRNA(0.846) |
| 27 | 5'-CC[A/T/C][A/T]GTGGA[G/A][C/T]T-3' | 1.00E-133 | -3.07E+02 | 1.19% | 0.44% | 100.2bp (199.4bp) | ZNF354C/MA0130.1/Jaspar(0.693) |
| 28 | 5'-GGGAGGCCTCAG-3' | 1.00E-131 | -3.04E+02 | 2.26% | 1.15% | 100.6bp (185.7bp) | HNRNPH2(RRM)/Homo_sapiens-RNCMPT00160-PBM/HughesRNA(0.649) |
| 29 | 5'-A[T/G/A]TGT[A/T/G]A[G/T]TTTC-3' | 1.00E-109 | -2.52E+02 | 3.84% | 2.46% | 103.9bp (156.1bp) | tll/dmmpmm(Papatsenko)/fly(0.698) |


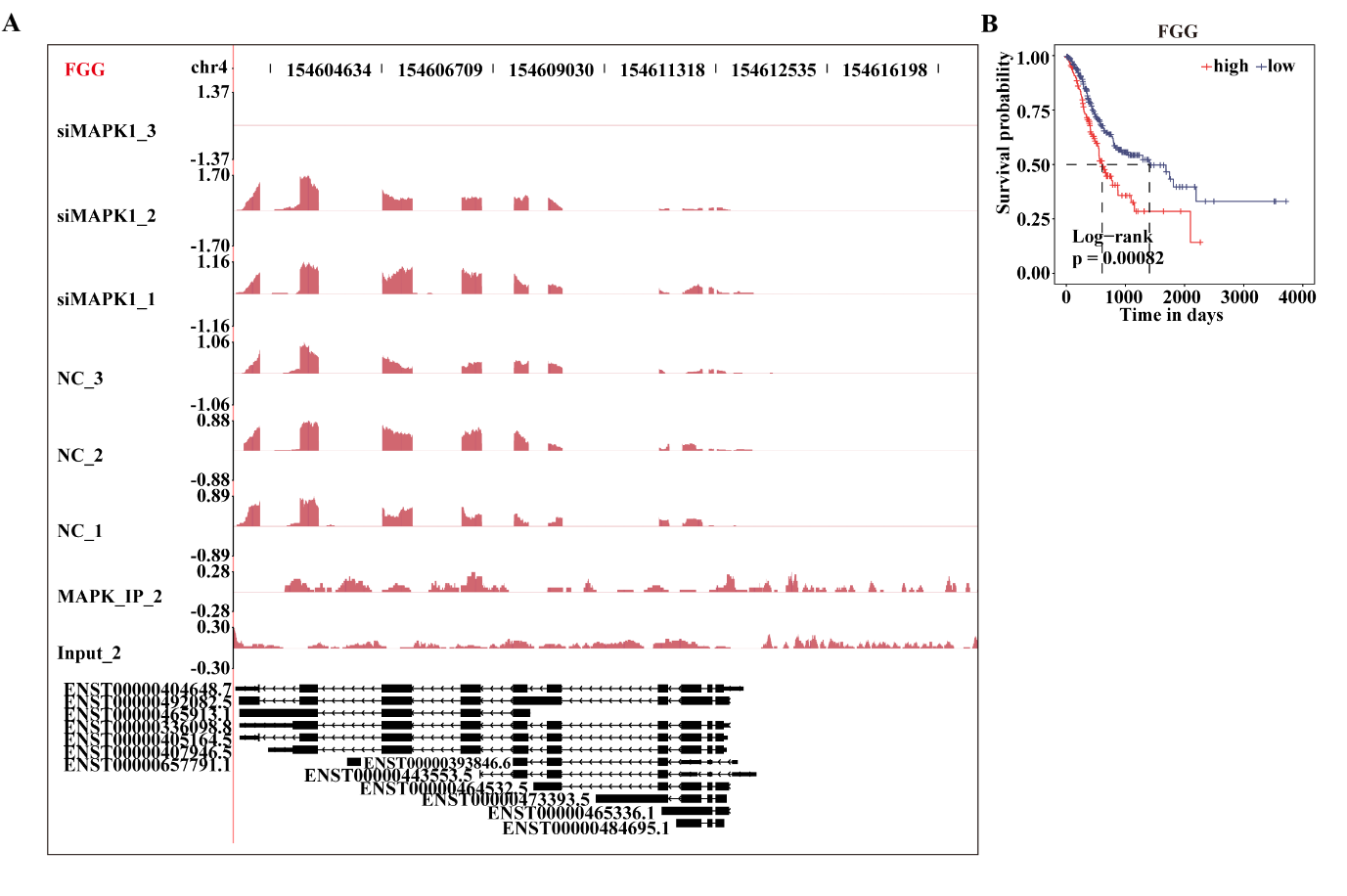


**Fig S1.** Validation of *FGG* associated with MAPK1. (**A**) The peak reads and binding locations throughout the mRNA are shown in the IGV Sashimi Plot. The red sections indicate the peak positions. The transcripts for each gene are shown below, and the read distribution of the bound gene is plotted in the top panel. (**B**) Prognostic line plots of a few significant genes from the TCGA data on gastric cancer. siMAPK1: short interfering MAPK1; NC: non-targeting control; Input: background noise without undergoing immunoprecipitation.


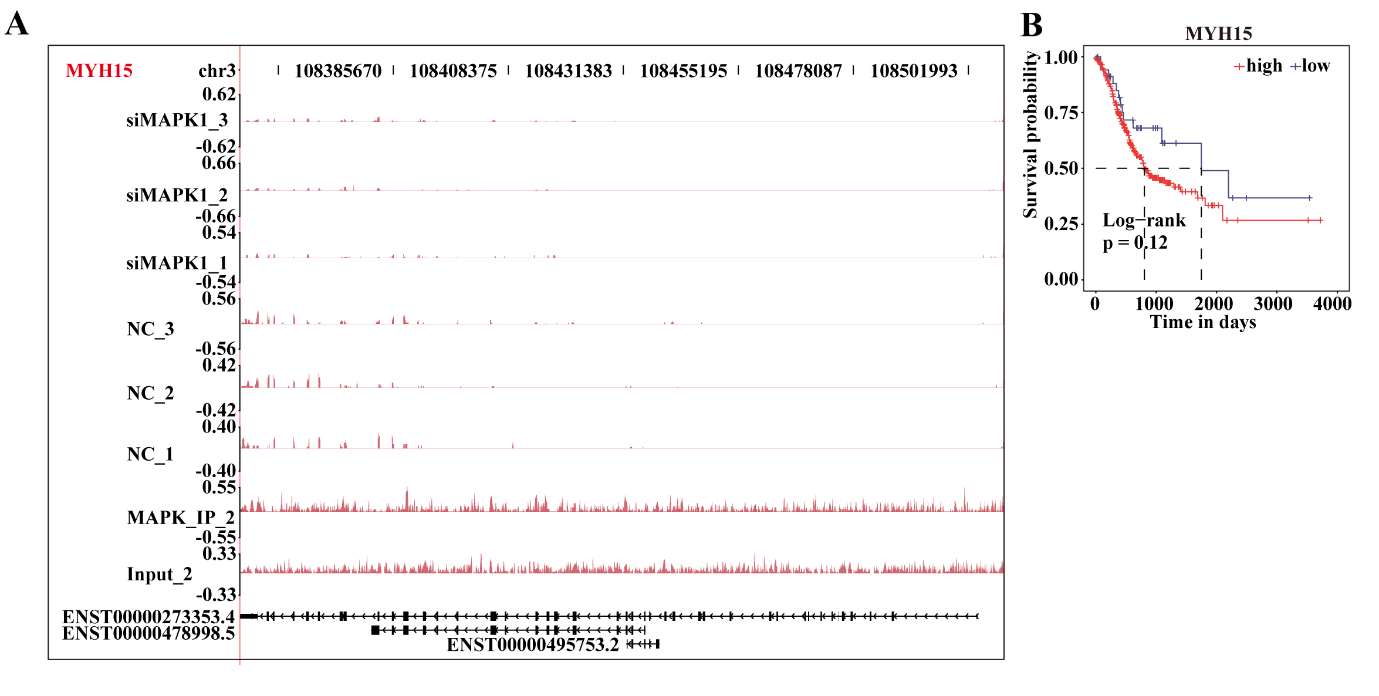


**Fig S2.** Validation of *MYH15* associated with MAPK1. (**A**) The peak reads and binding locations throughout the mRNA are shown in the IGV Sashimi Plot. The red sections indicate the peak positions. The transcripts for each gene are shown below, and the read distribution of the bound gene is plotted in the top panel. (**B**) Prognostic line plots of a few significant genes from the TCGA data on gastric cancer. siMAPK1: short interfering MAPK1; NC: non-targeting control; Input: background noise without undergoing immunoprecipitation.


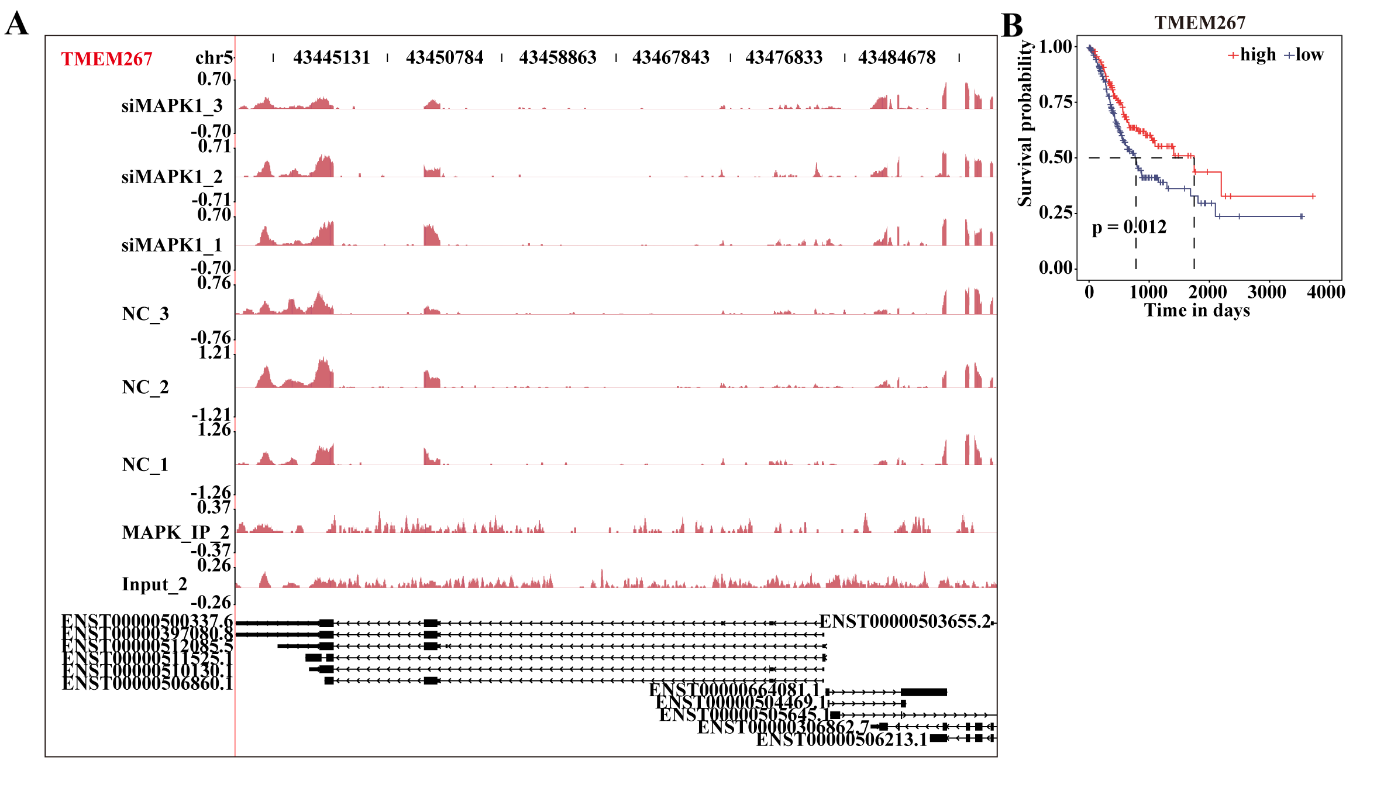


**Fig S3.** Validation of *TMEM267* associated with MAPK1. (**A**) The peak reads and binding locations throughout the mRNA are shown in the IGV Sashimi Plot. The red sections indicate the peak positions. The transcripts for each gene are shown below, and the read distribution of the bound gene is plotted in the top panel. (**B**) Prognostic line plots of a few significant genes from the TCGA data on gastric cancer. siMAPK1: short interfering MAPK1; NC: non-targeting control; Input: background noise without undergoing immunoprecipitation.


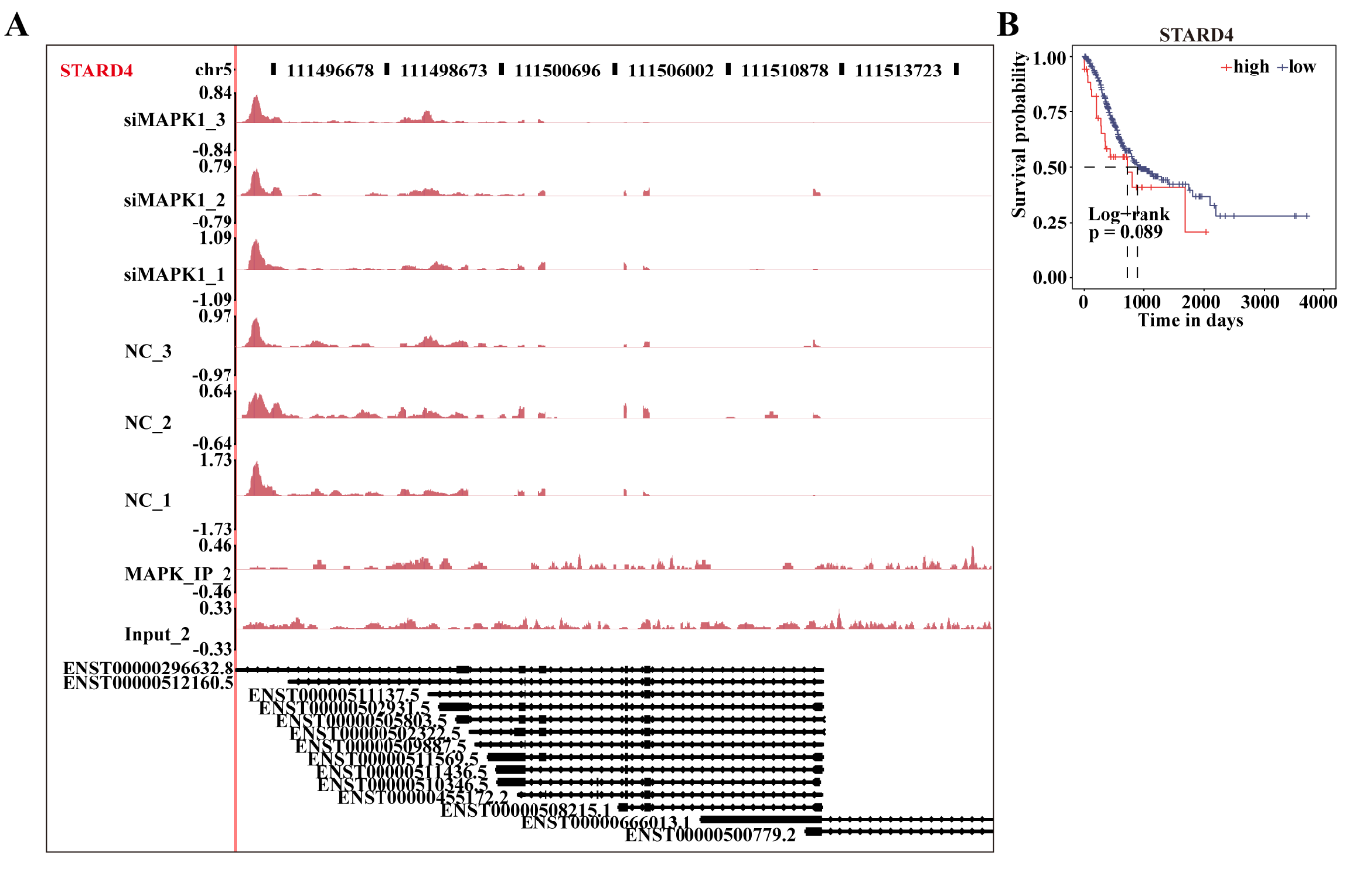


**Fig S4.** Validation of *STARD4* associated with MAPK1. (**A**) The peak reads and binding locations throughout the mRNA are shown in the IGV Sashimi Plot. The red sections indicate the peak positions. The transcripts for each gene are shown below, and the read distribution of the bound gene is plotted in the top panel. (**B**) Prognostic line plots of a few significant genes from the TCGA data on gastric cancer. siMAPK1: short interfering MAPK1; NC: non-targeting control; Input: background noise without undergoing immunoprecipitation.


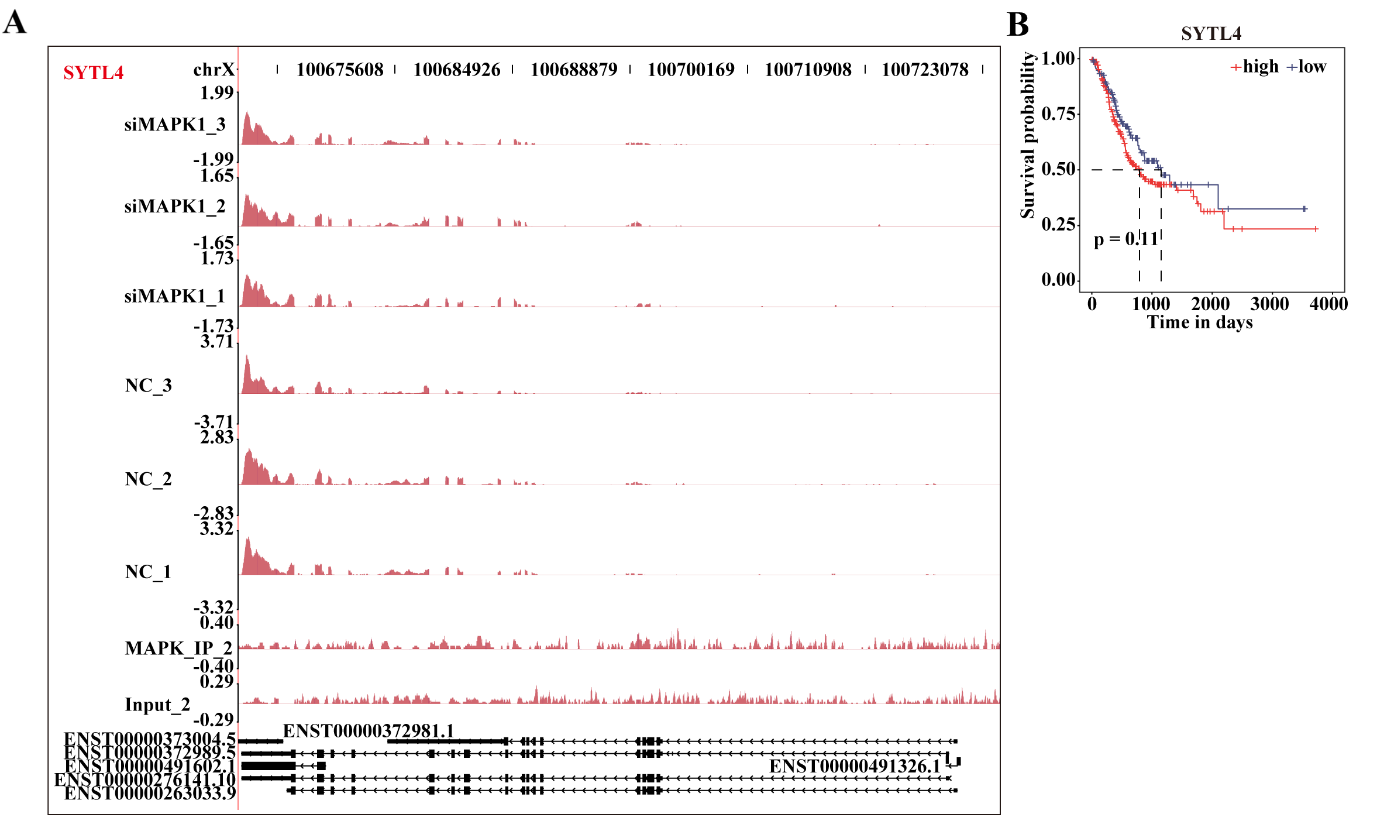


**Fig S5.** Validation of *SYTL4* associated with MAPK1. (**A**) The peak reads and binding locations throughout the mRNA are shown in the IGV Sashimi Plot. The red sections indicate the peak positions. The transcripts for each gene are shown below, and the read distribution of the bound gene is plotted in the top panel. (**B**) Prognostic line plots of a few significant genes from the TCGA data on gastric cancer. siMAPK1: short interfering MAPK1; NC: non-targeting control; Input: background noise without undergoing immunoprecipitation.
