# Supplementary material for: MAPK1 promotes the metastasis and invasion of gastric cancer as a bidirectional transcription factor
Source: BMC Cancer. 2023 Oct 10;23:959. doi: 10.1186/s12885-023-11480-3 (PMC10563293; doi:10.1186/s12885-023-11480-3)
Supplement: Supplementary file 2 — Supplementary Material 2 [file 12885_2023_11480_MOESM2_ESM.docx]

**Supplementary Material 2.** **Full-length gels and blots**


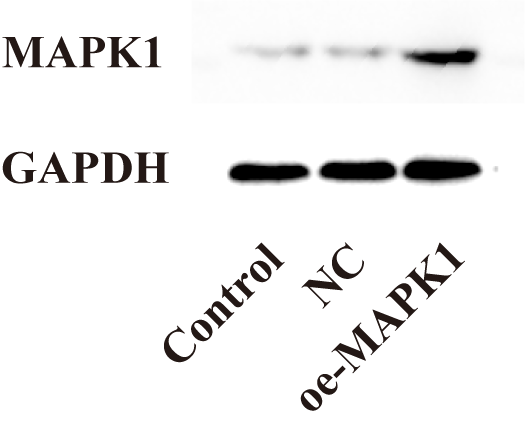


**Full-length and multiple exposures of gels and blots of Fig 1B**

**
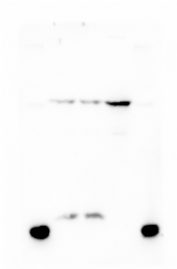

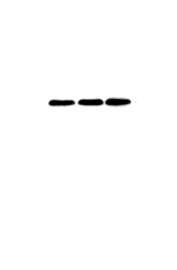
**

NC

oe-MAPK1

NC

Control

oe-MAPK1

Control

GAPDH

MAPK1

**
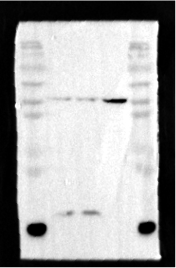

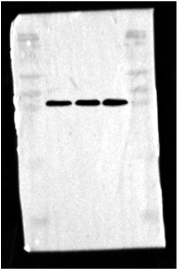
**

NC

oe-MAPK1

oe-MAPK1

Control

NC

Control

GAPDH

MAPK1

**
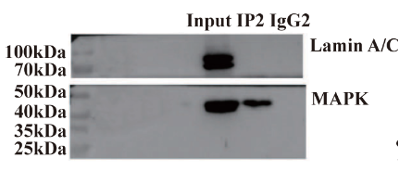
**

**Full-length of gels and blots for Fig 3A**

**
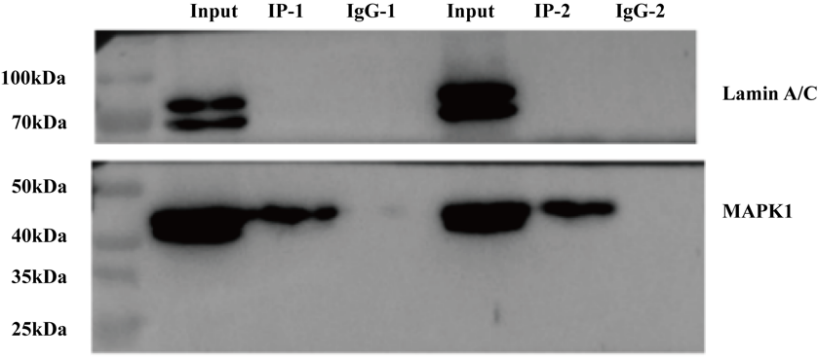
**
